# Supplementary figures and images for: Srs2 binding to proliferating cell nuclear antigen (PCNA) and its sumoylation contribute to replication protein A (RPA) antagonism during the DNA damage response
Source: eLife. 2025 Aug 1;13:RP98843. doi: 10.7554/eLife.98843 (PMC12316459; doi:10.7554/eLife.98843)

black squares indicate the lanes that showed in figure

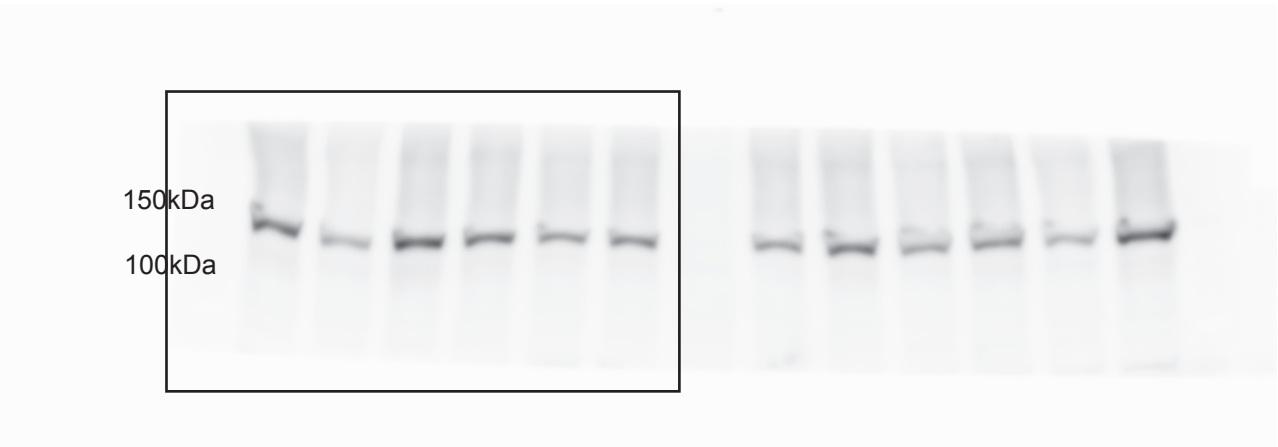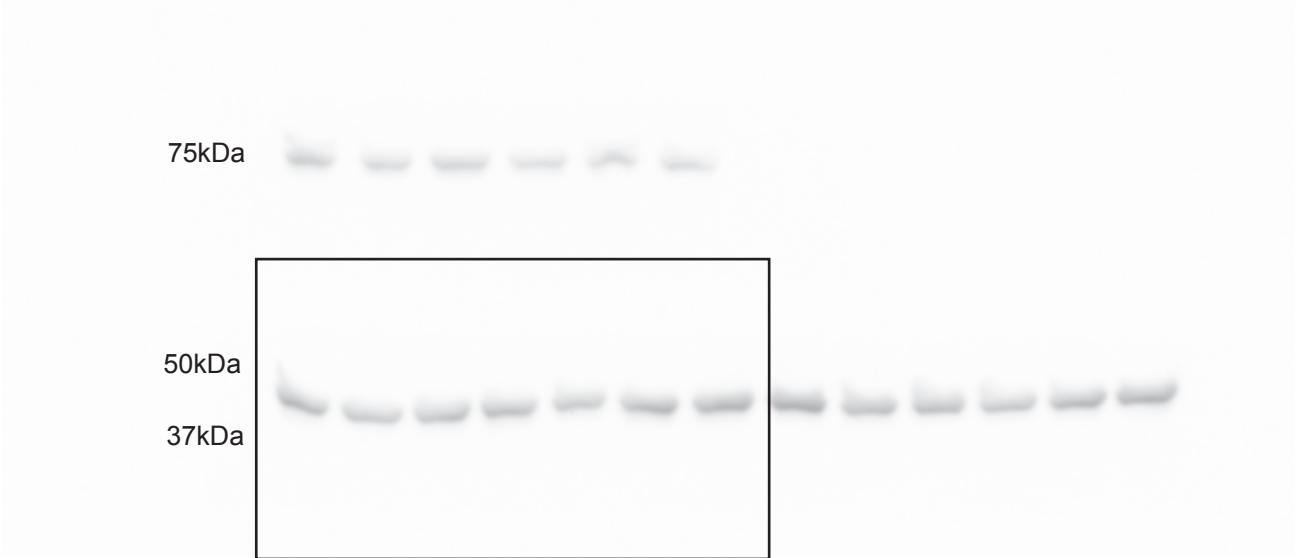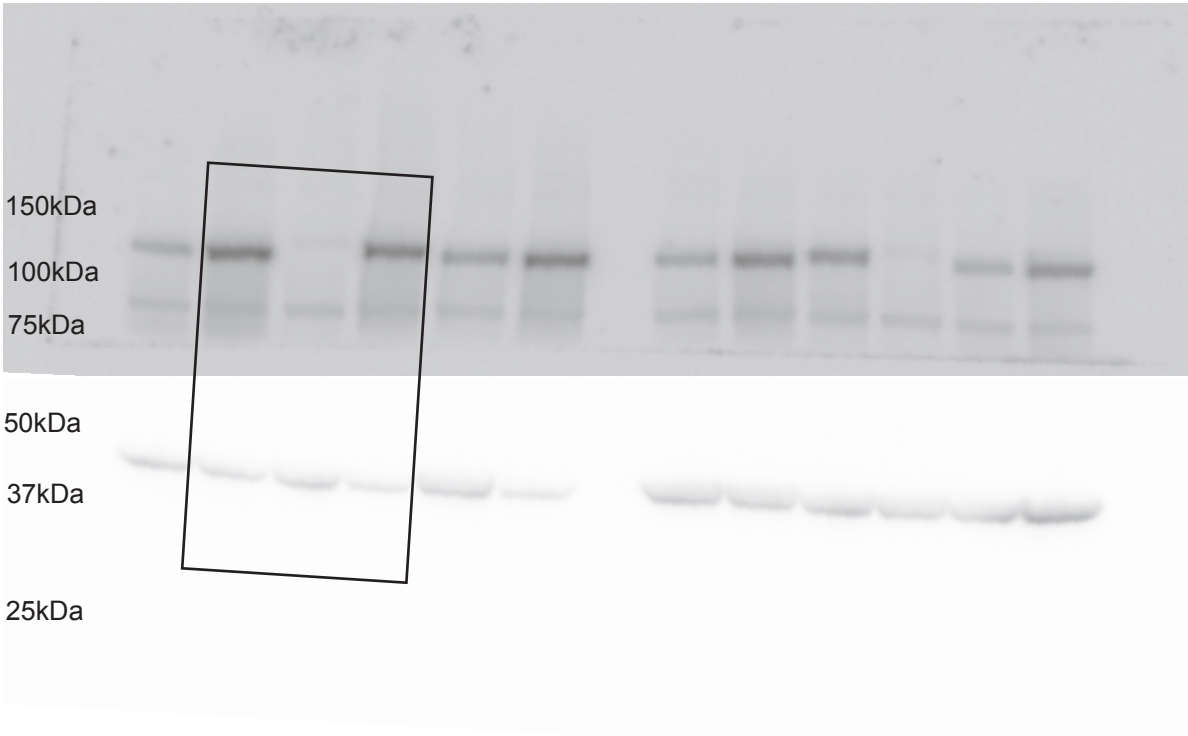

Supplement: Figure 1—source data 1. [file elife-98843-fig1-data1.pdf]

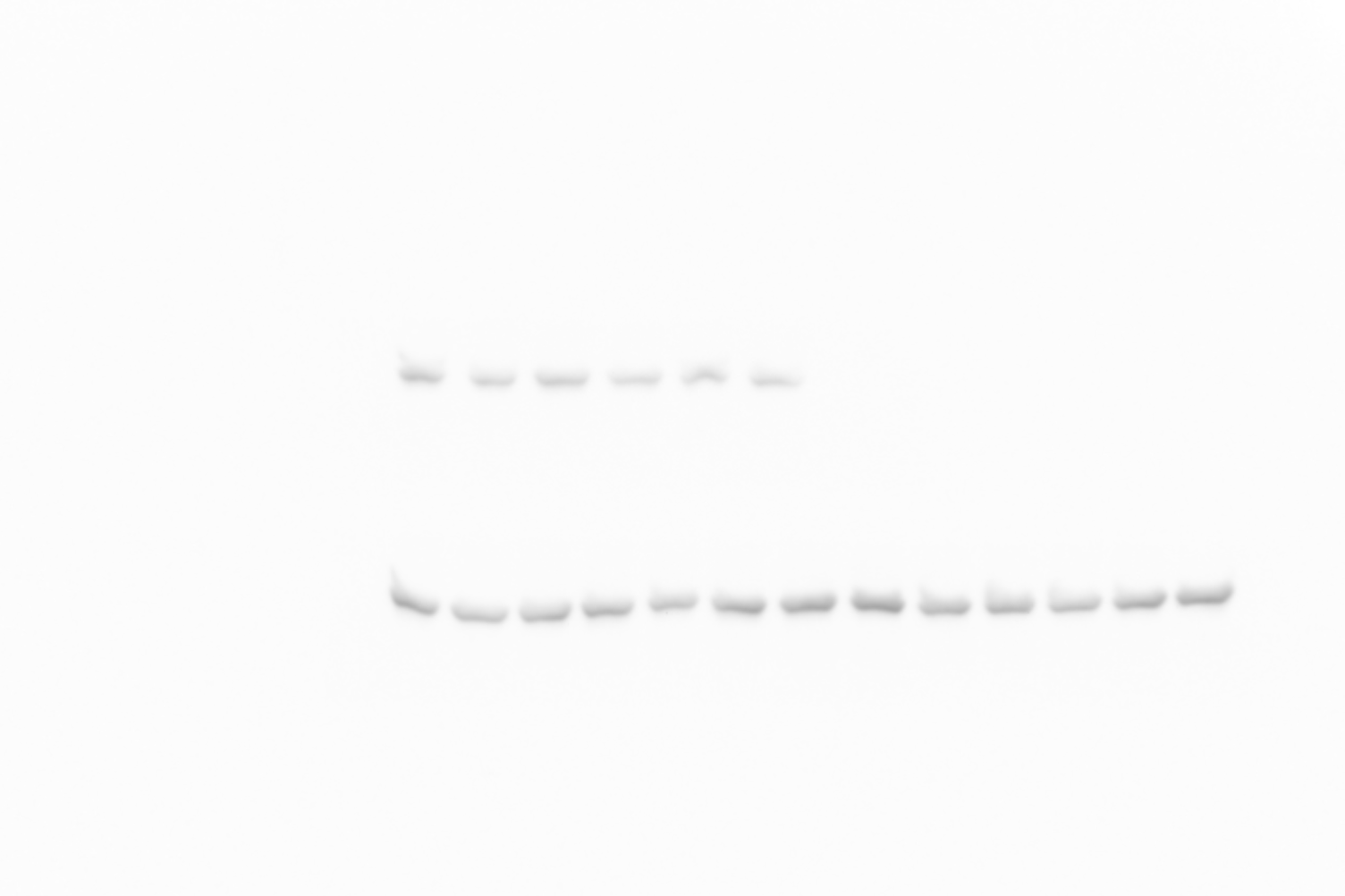

Supplement: Figure 1—source data 2. [file elife-98843-fig1-data2.zip › Pgk1 left part.tif]

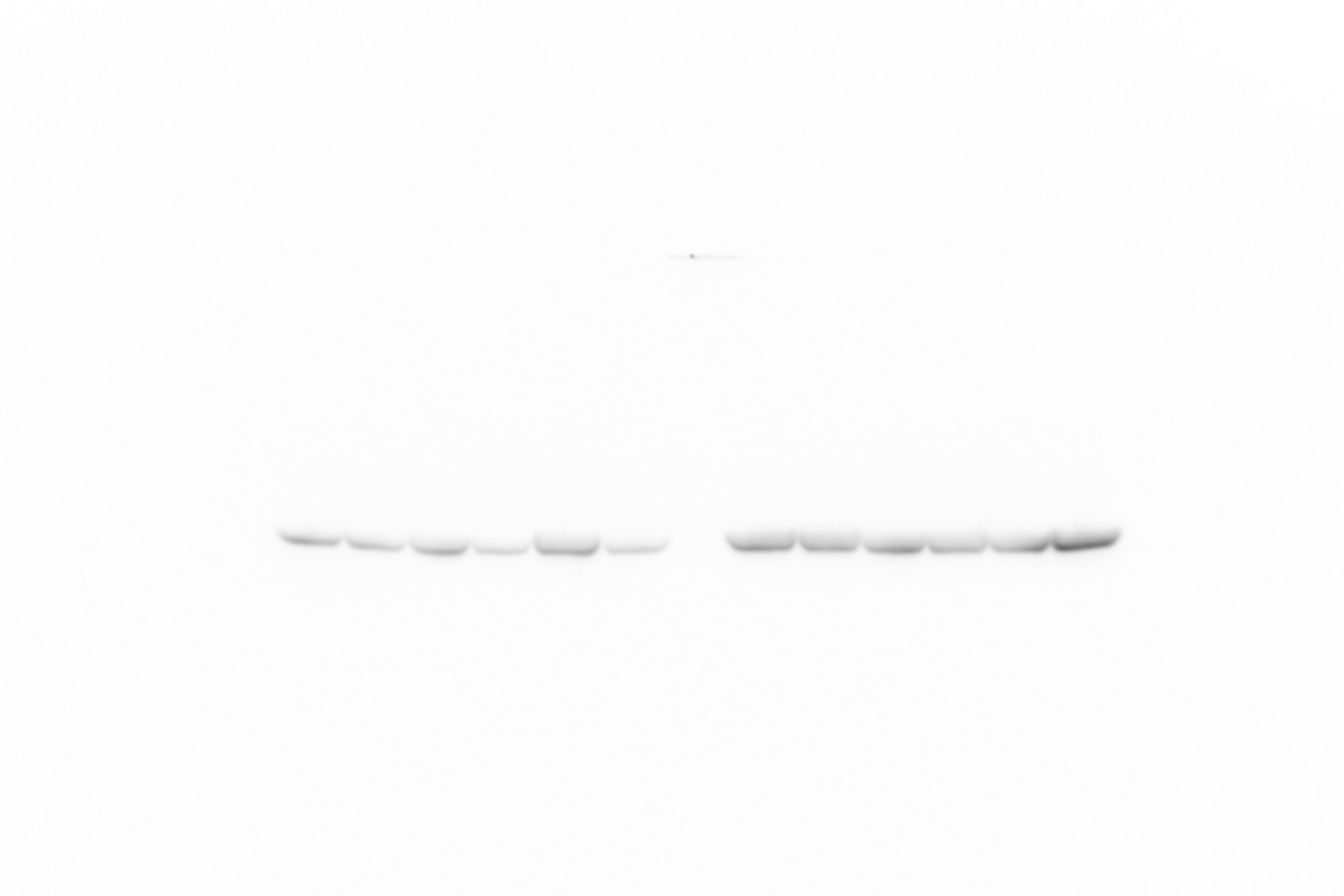

Supplement: Figure 1—source data 2. [file elife-98843-fig1-data2.zip › Pgk1 right part.tif]

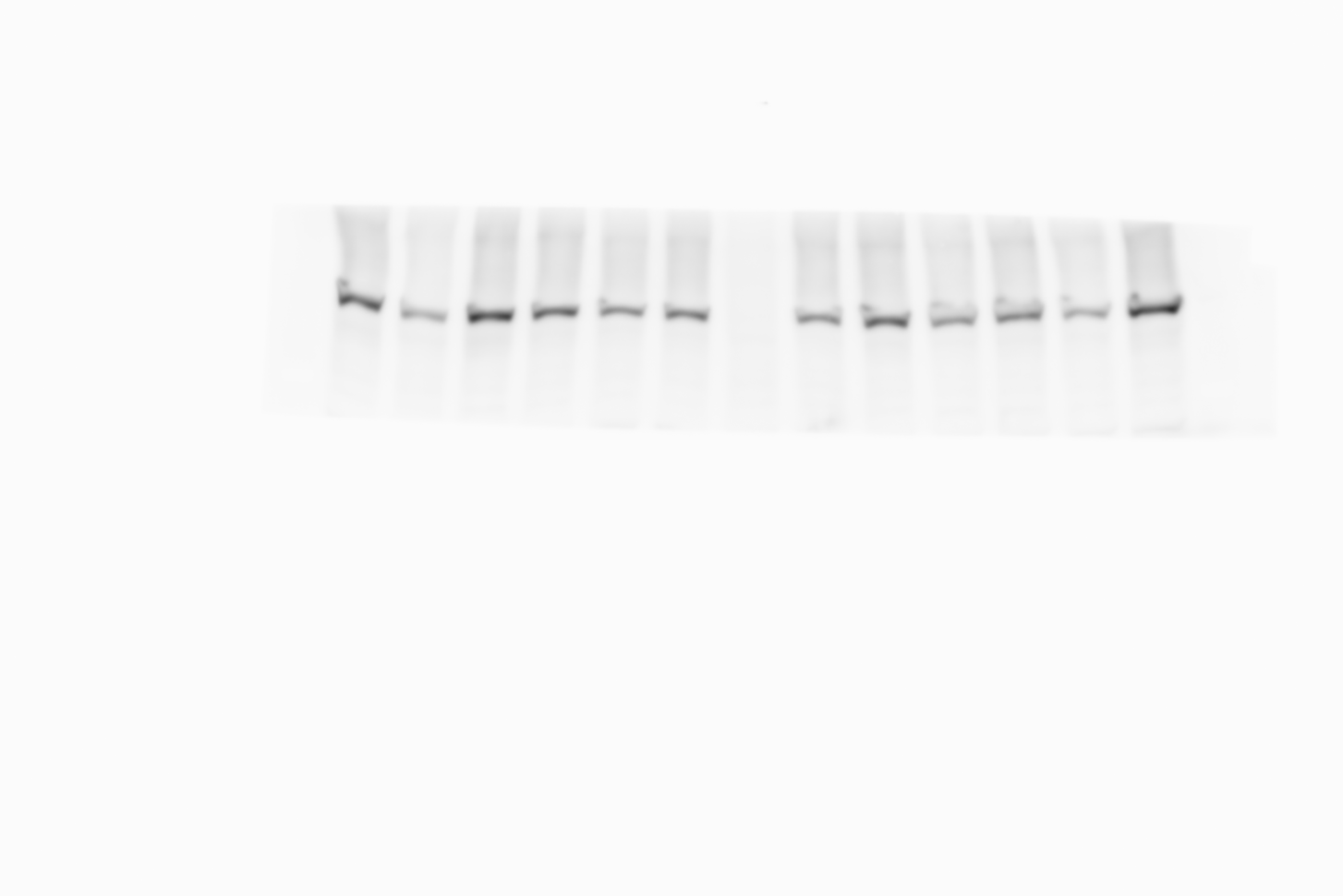

Supplement: Figure 1—source data 2. [file elife-98843-fig1-data2.zip › Srs2 left part.tif]

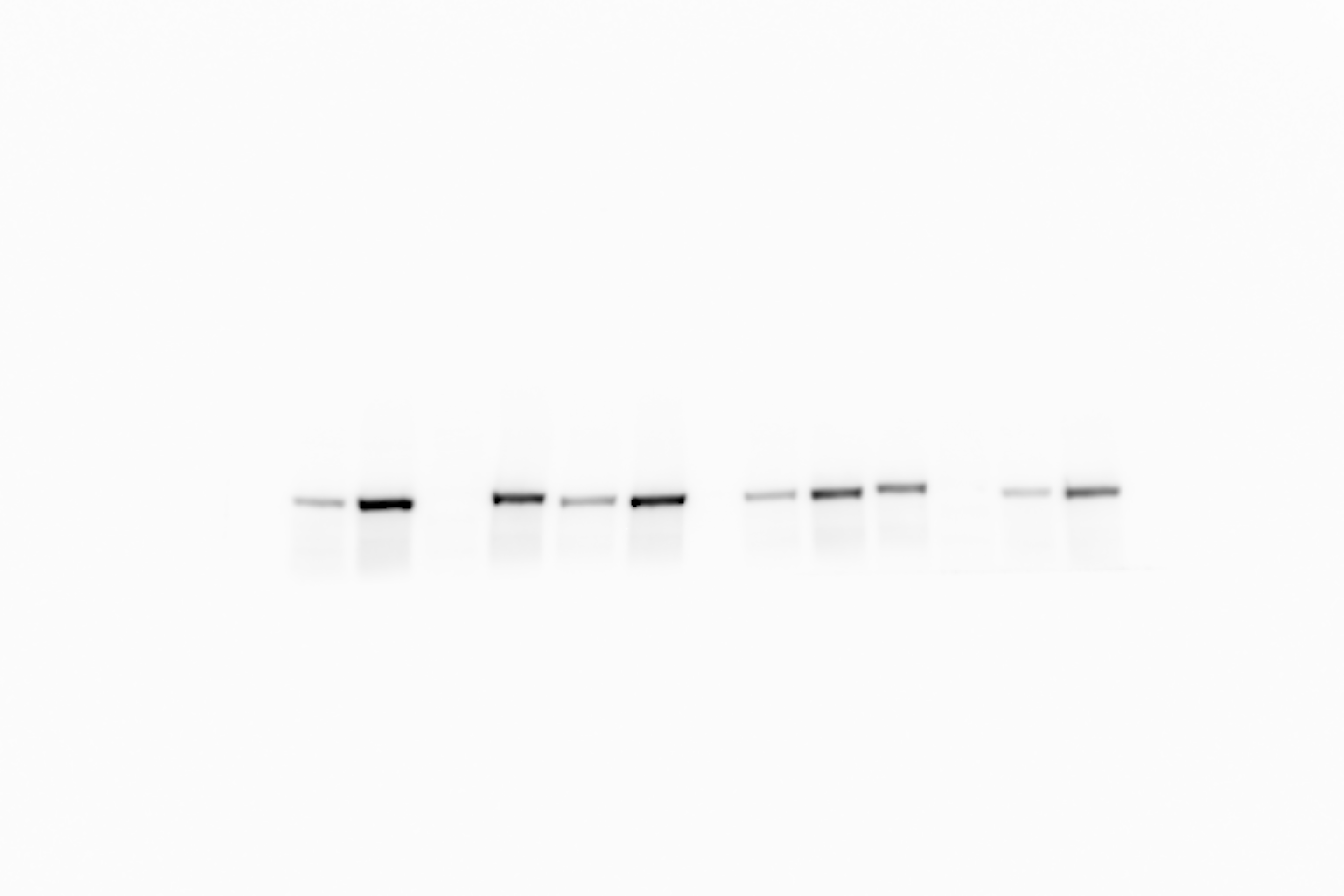

Supplement: Figure 1—source data 2. [file elife-98843-fig1-data2.zip › Srs2 right part.tif]

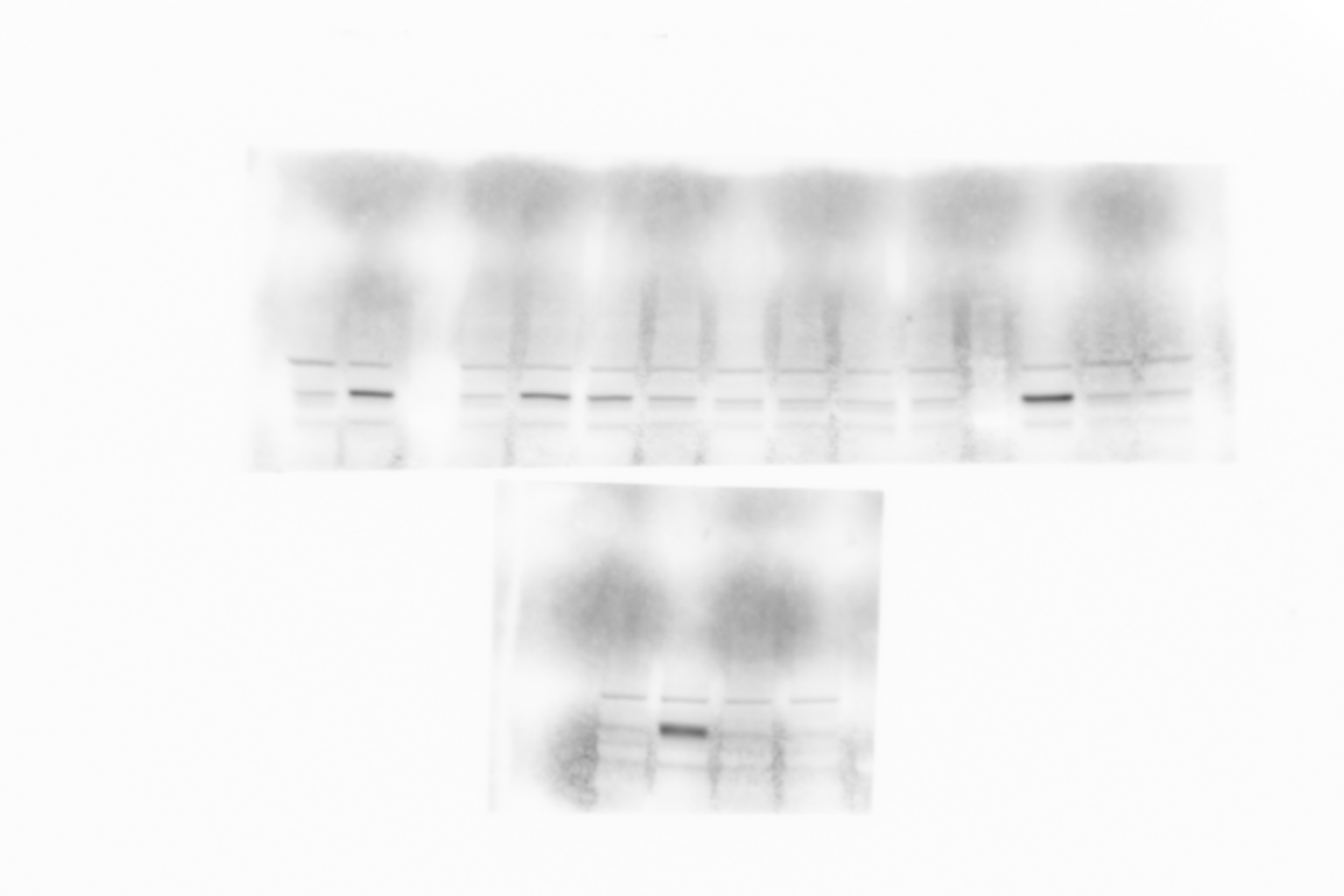

Supplement: Figure 3—source data 2. [file elife-98843-fig3-data2.zip › F9_Scanner.tif]

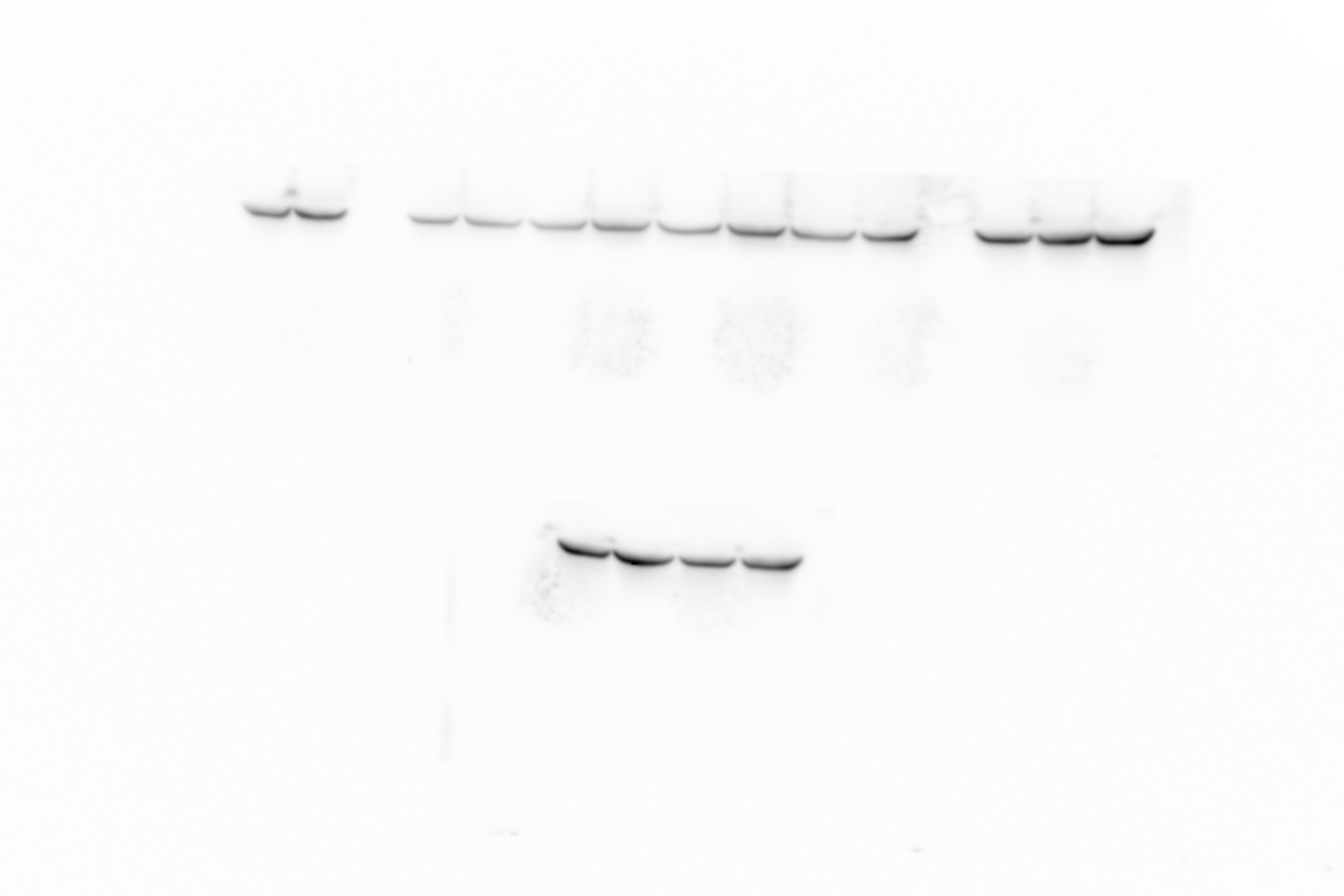

Supplement: Figure 3—source data 2. [file elife-98843-fig3-data2.zip › Pgk1_Scanner.tif]

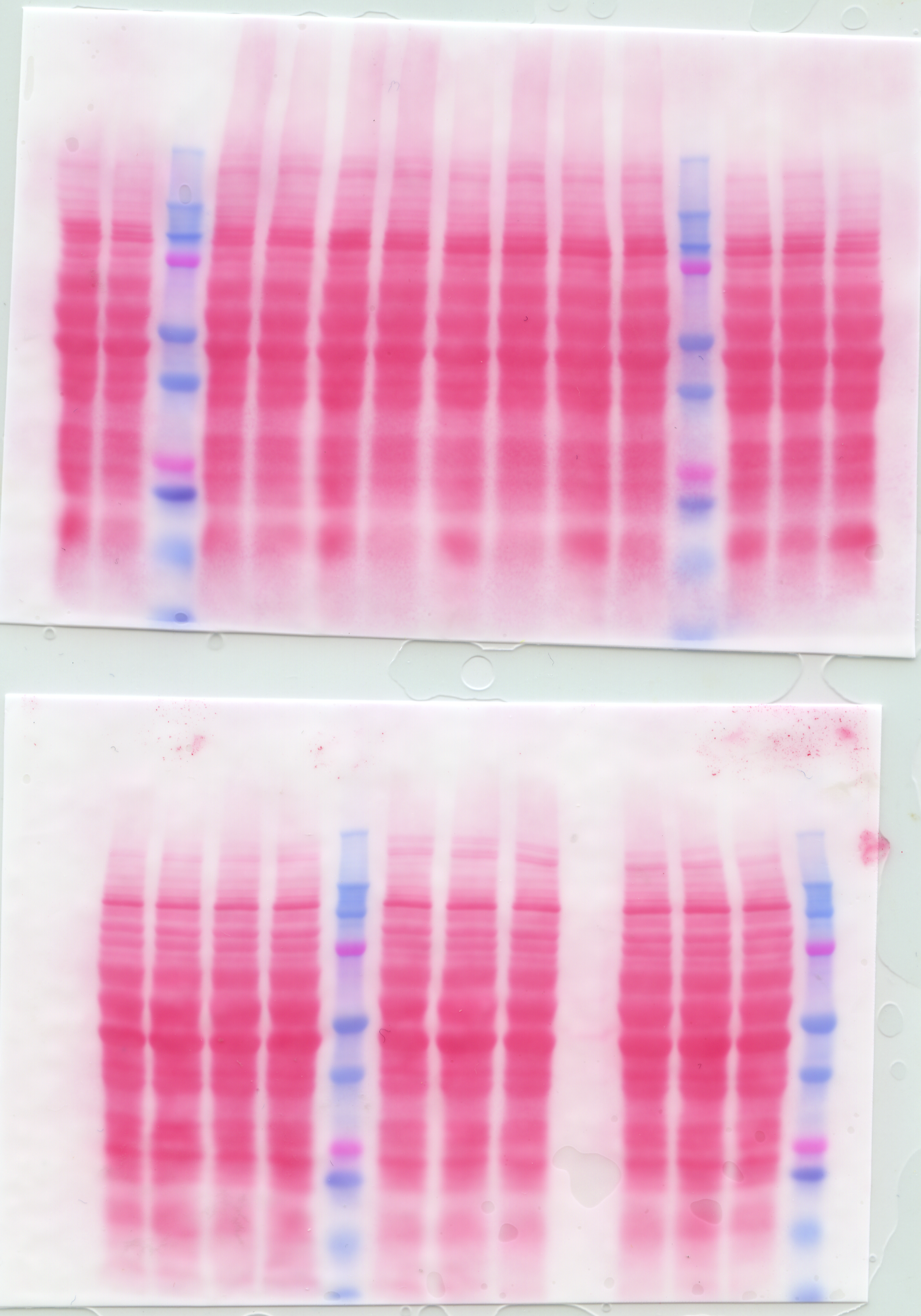

Supplement: Figure 3—source data 2. [file elife-98843-fig3-data2.zip › Ponceau Staining.tif]

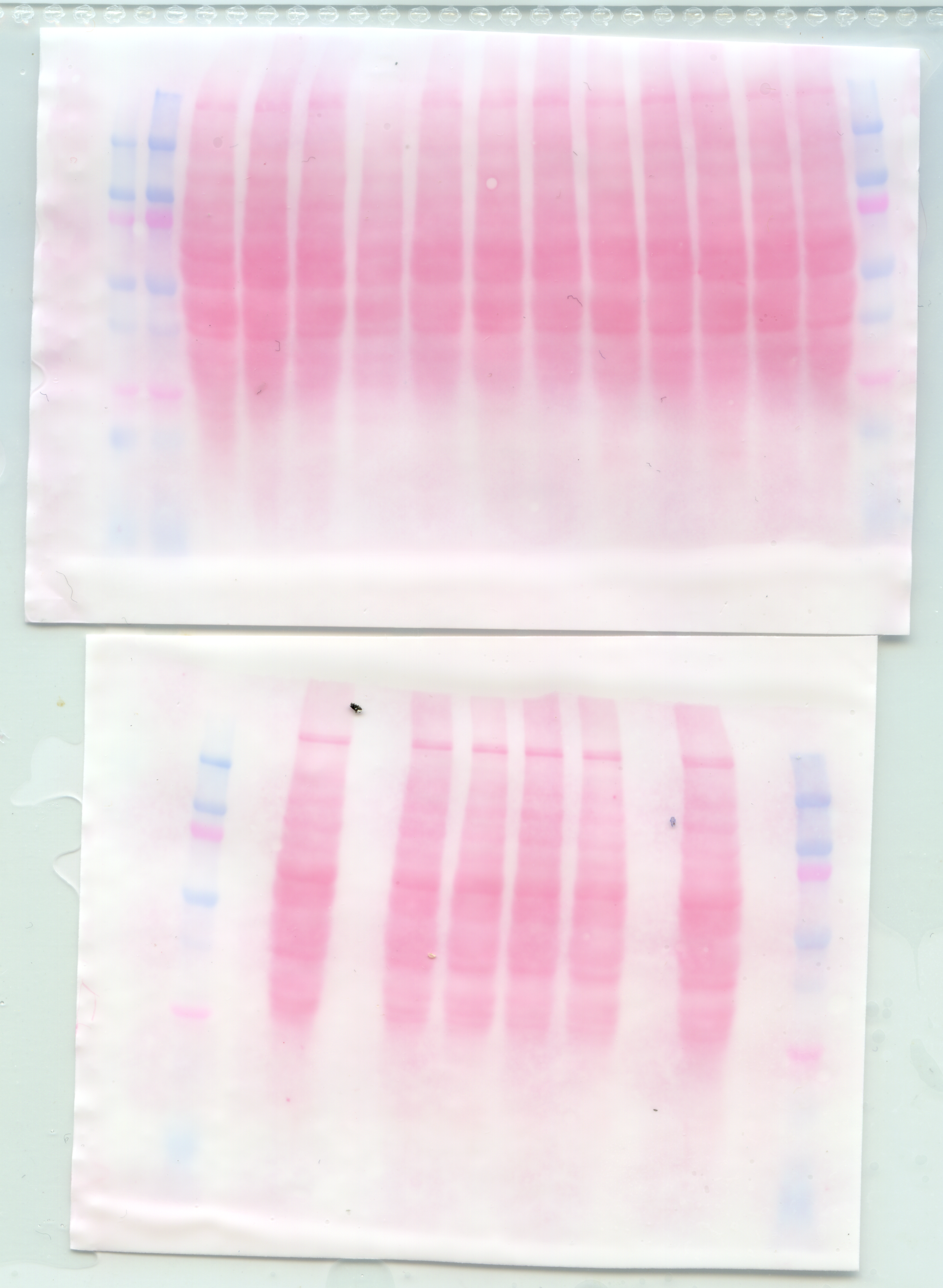

Supplement: Figure 4—source data 2. [file elife-98843-fig4-data2.zip › Ponceau Staining.tif]

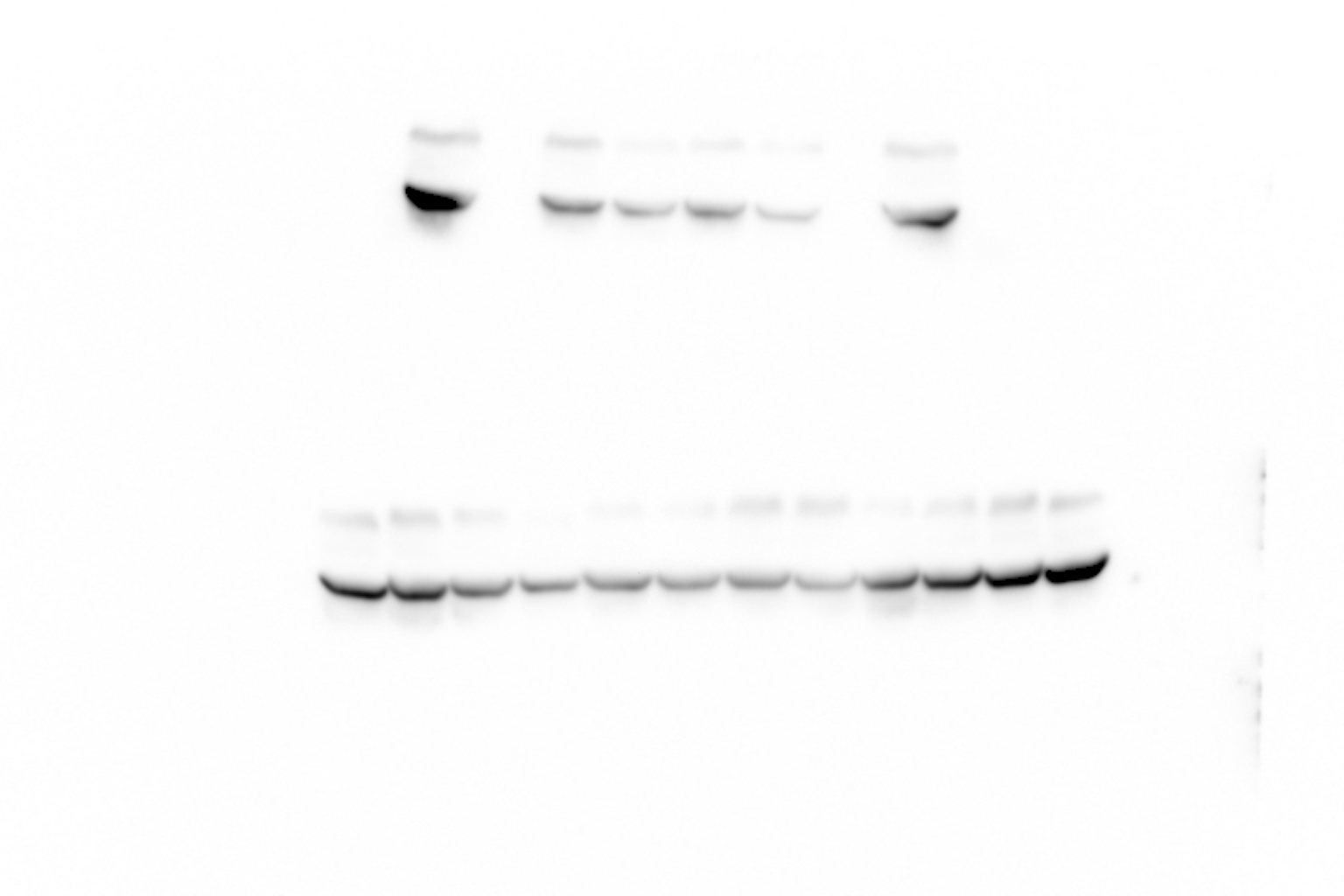

Supplement: Figure 4—source data 2. [file elife-98843-fig4-data2.zip › Pgk1_scanner.tif]

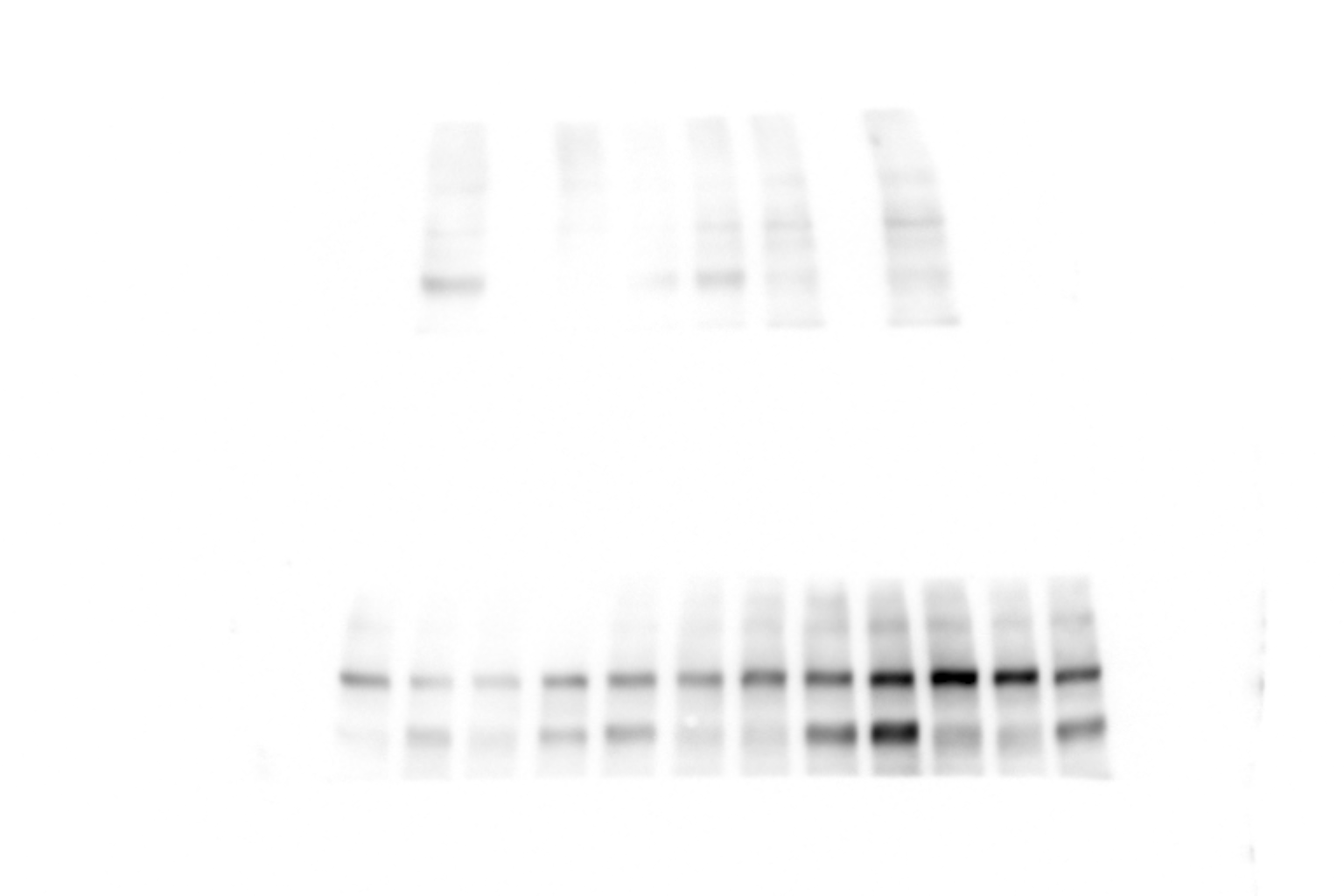

Supplement: Figure 4—source data 2. [file elife-98843-fig4-data2.zip › F9_Scanner.tif]

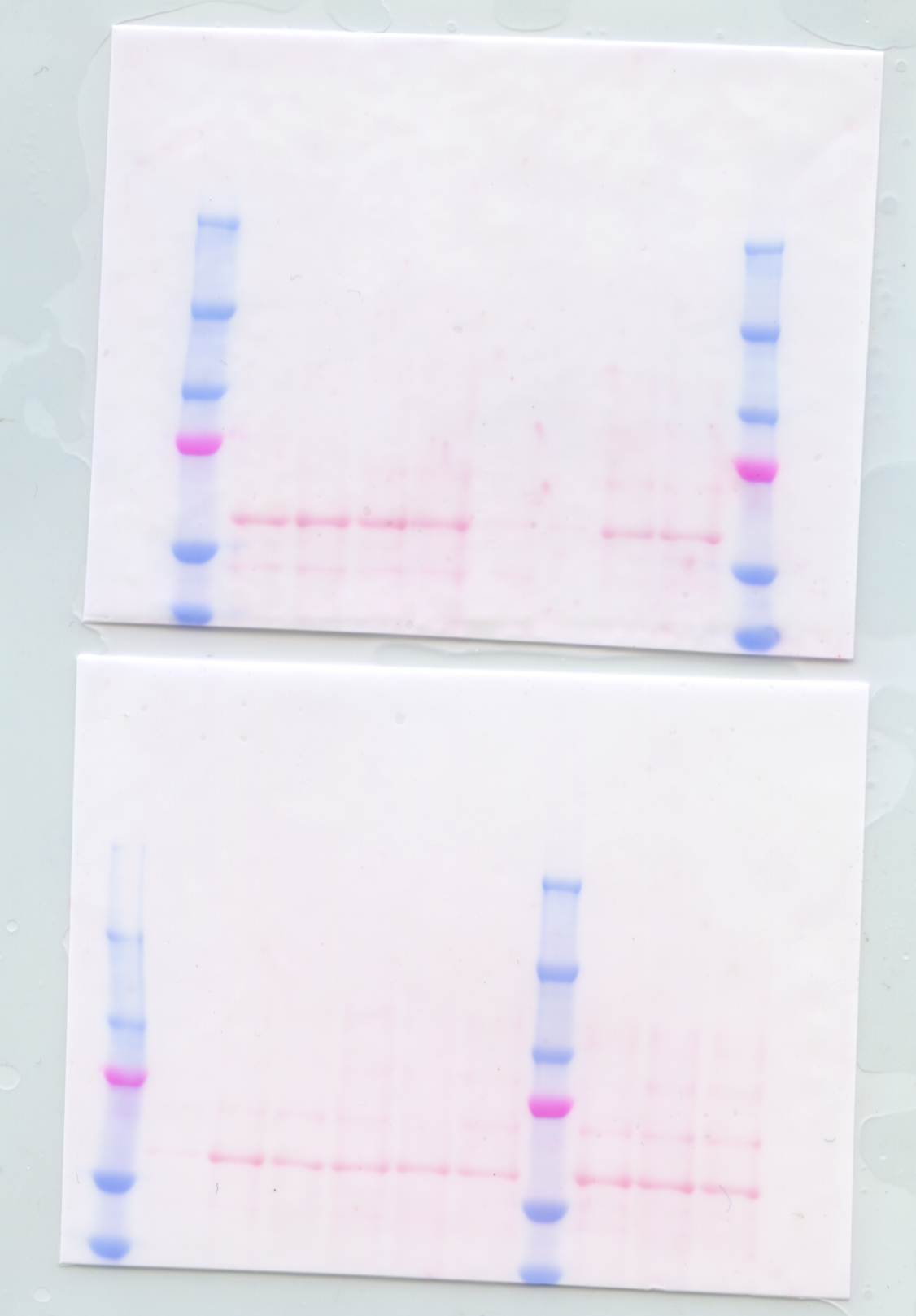

Supplement: Figure 5—source data 2. [file elife-98843-fig5-data2.zip › 5A Ponceau Staining.tif]

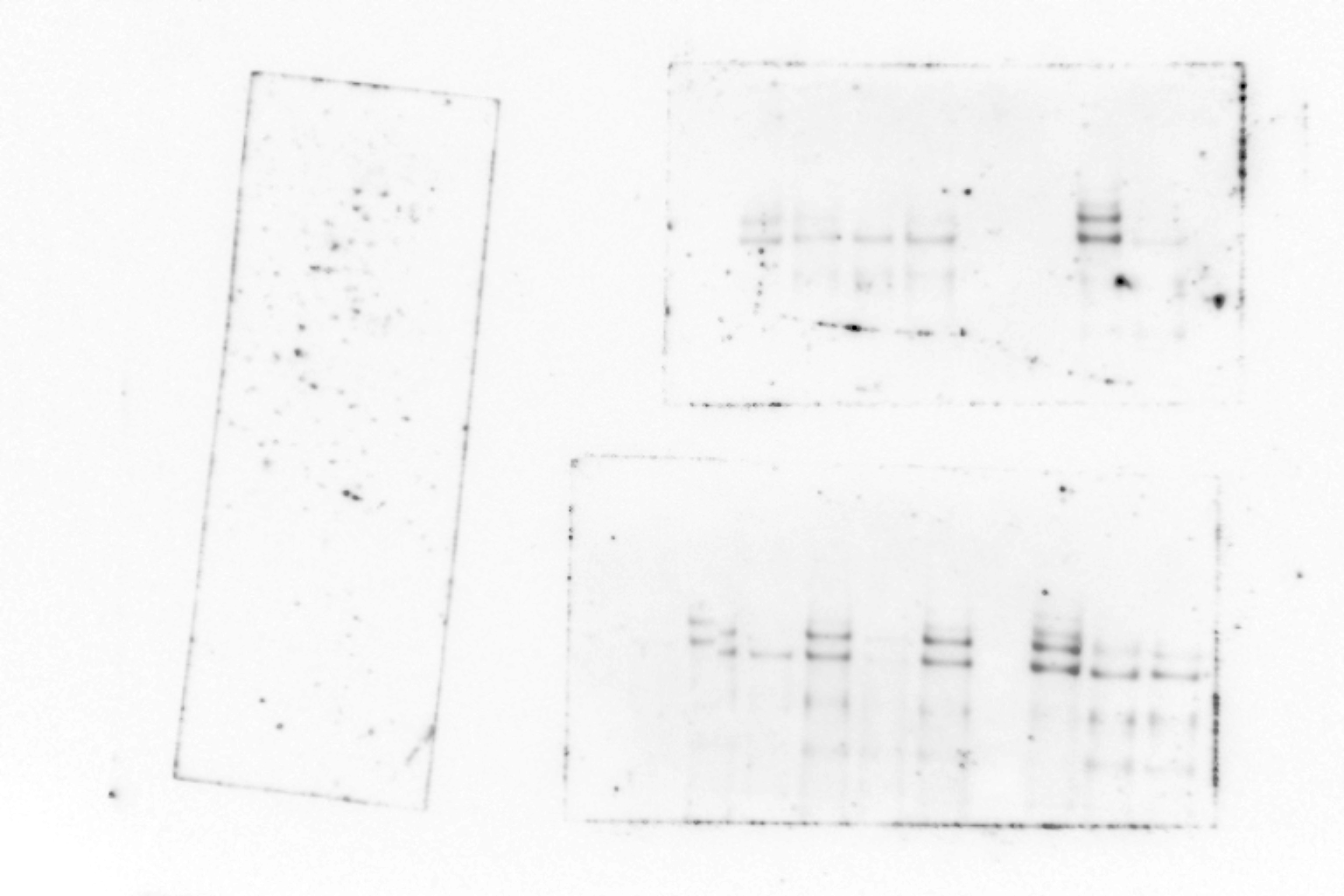

Supplement: Figure 5—source data 2. [file elife-98843-fig5-data2.zip › 5A Srs2_Scanner.tif]

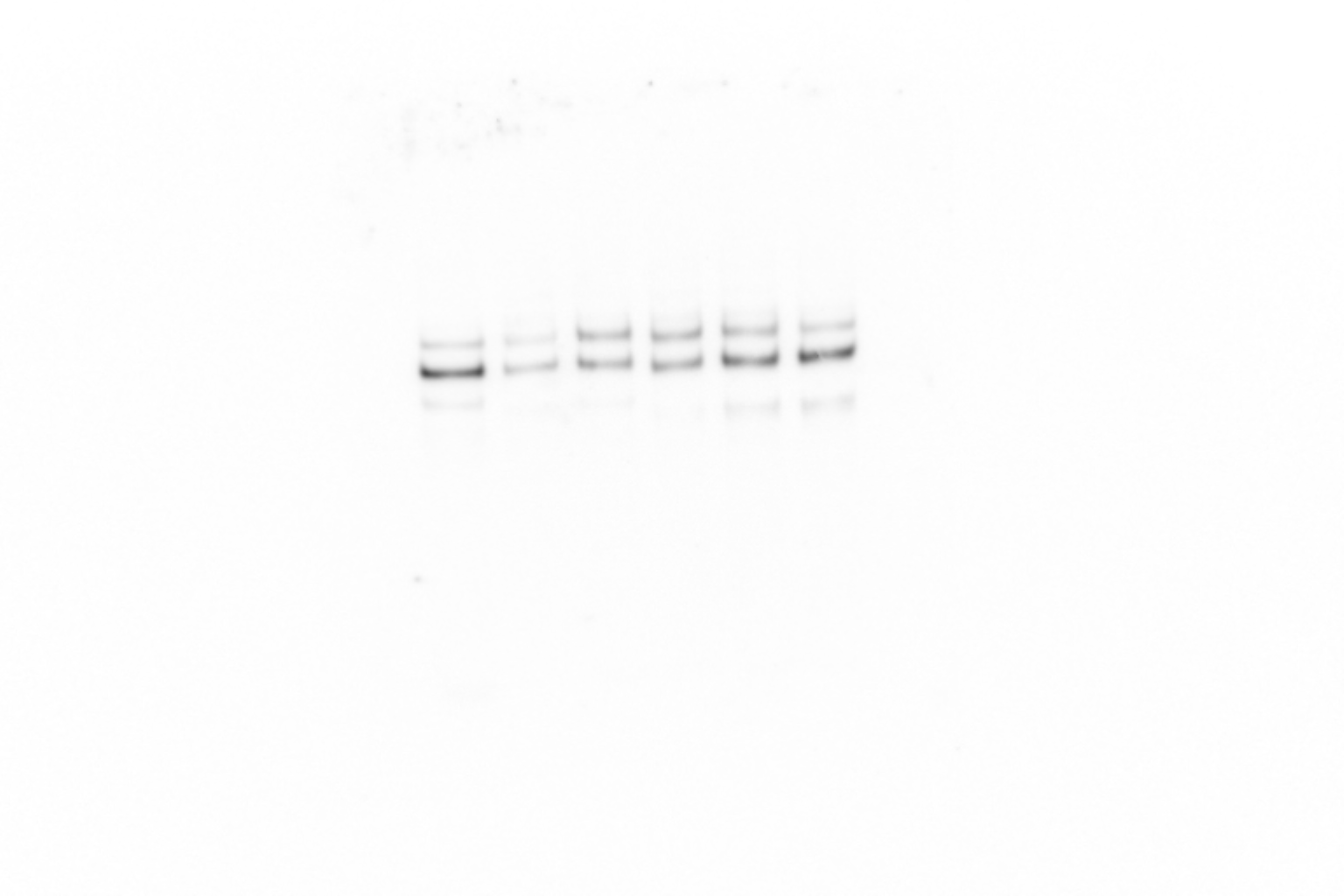

Supplement: Figure 5—source data 3. [file elife-98843-fig5-data3.zip › 5B Srs2_Scanner.tif]

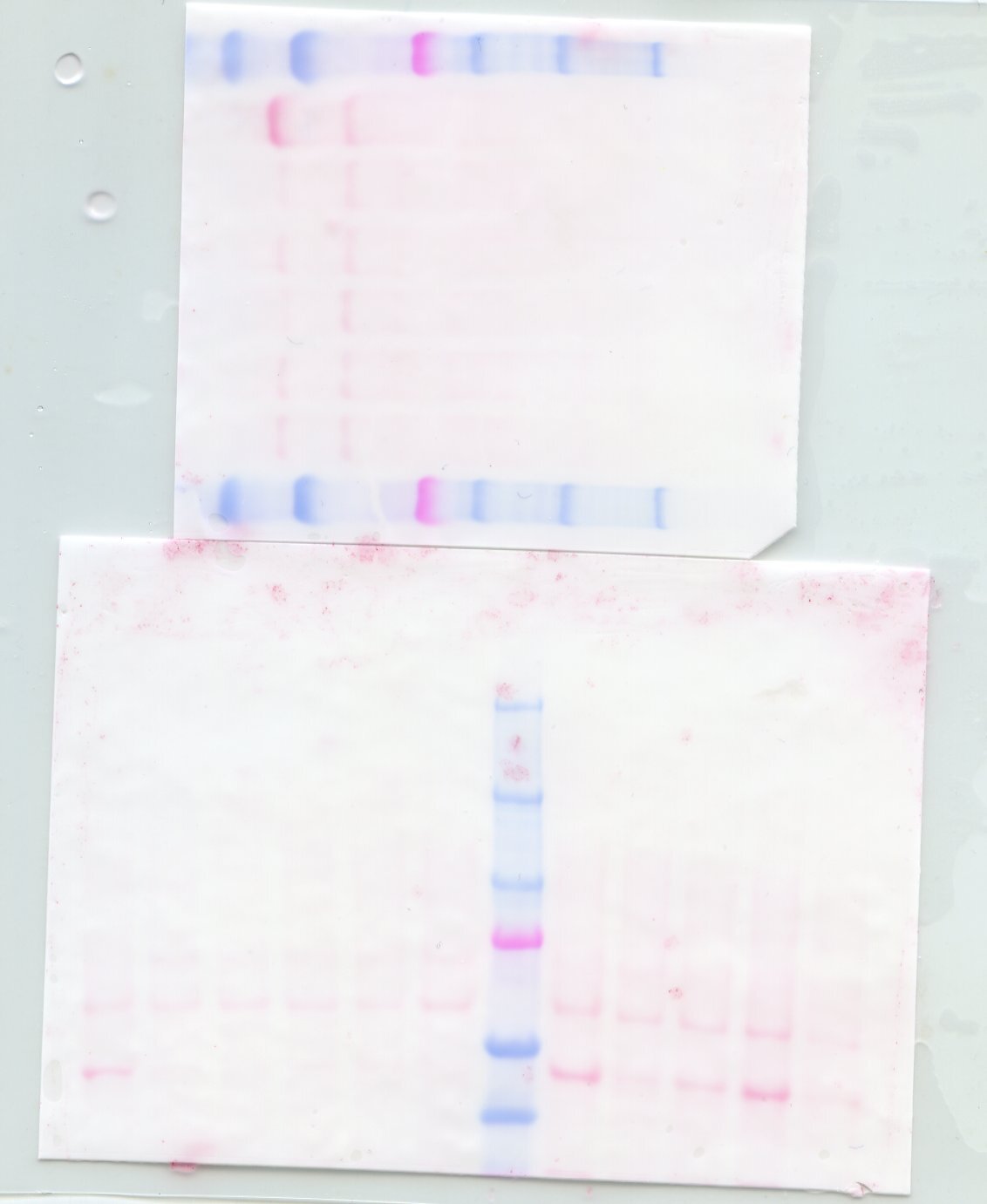

Supplement: Figure 5—source data 3. [file elife-98843-fig5-data3.zip › 5B Ponceau Staining.jpg]

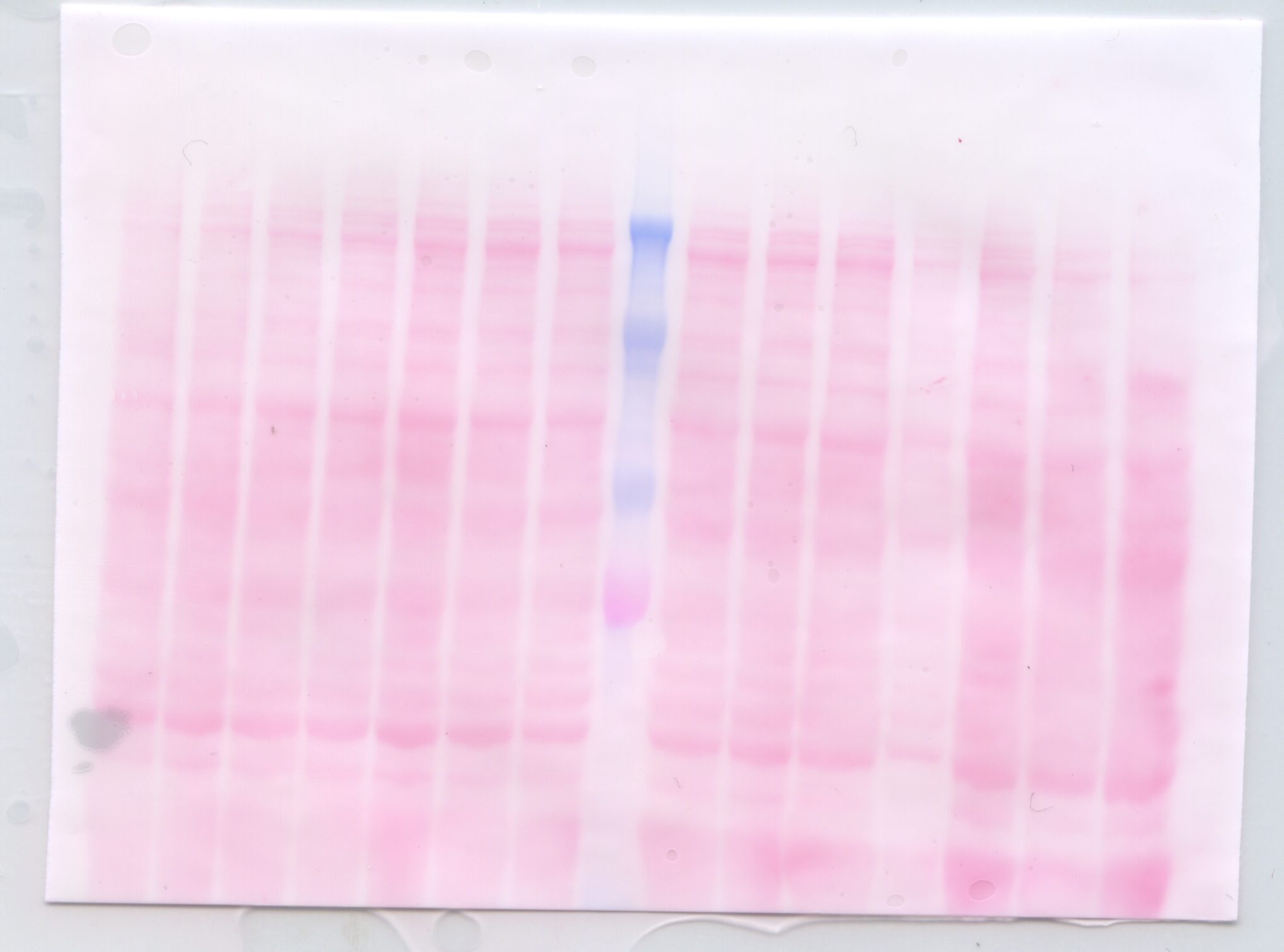

Supplement: Figure 5—source data 4. [file elife-98843-fig5-data4.zip › 5C Ponceau Staining.tif]

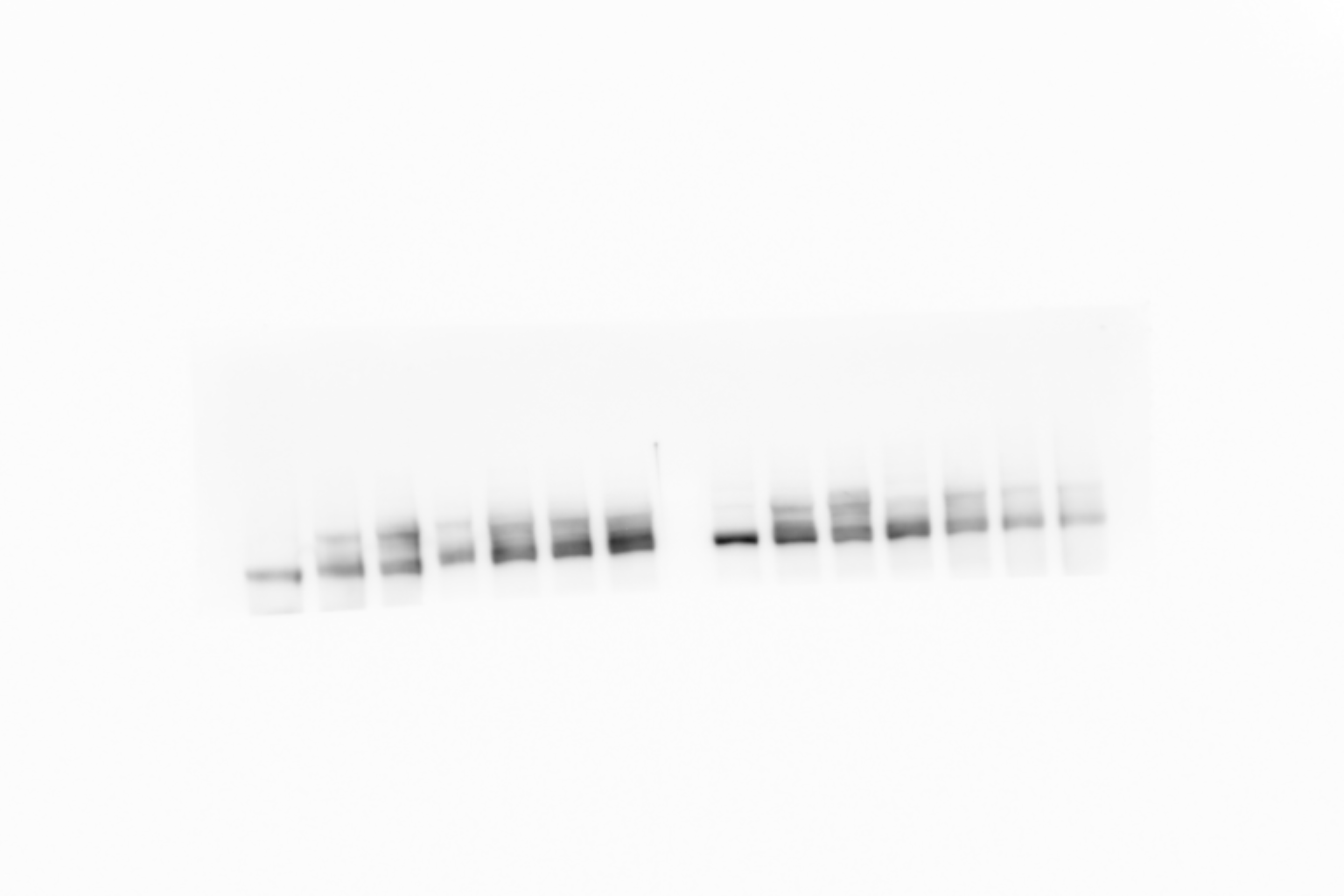

Supplement: Figure 5—source data 4. [file elife-98843-fig5-data4.zip › 5C rad9_scanner.tif]

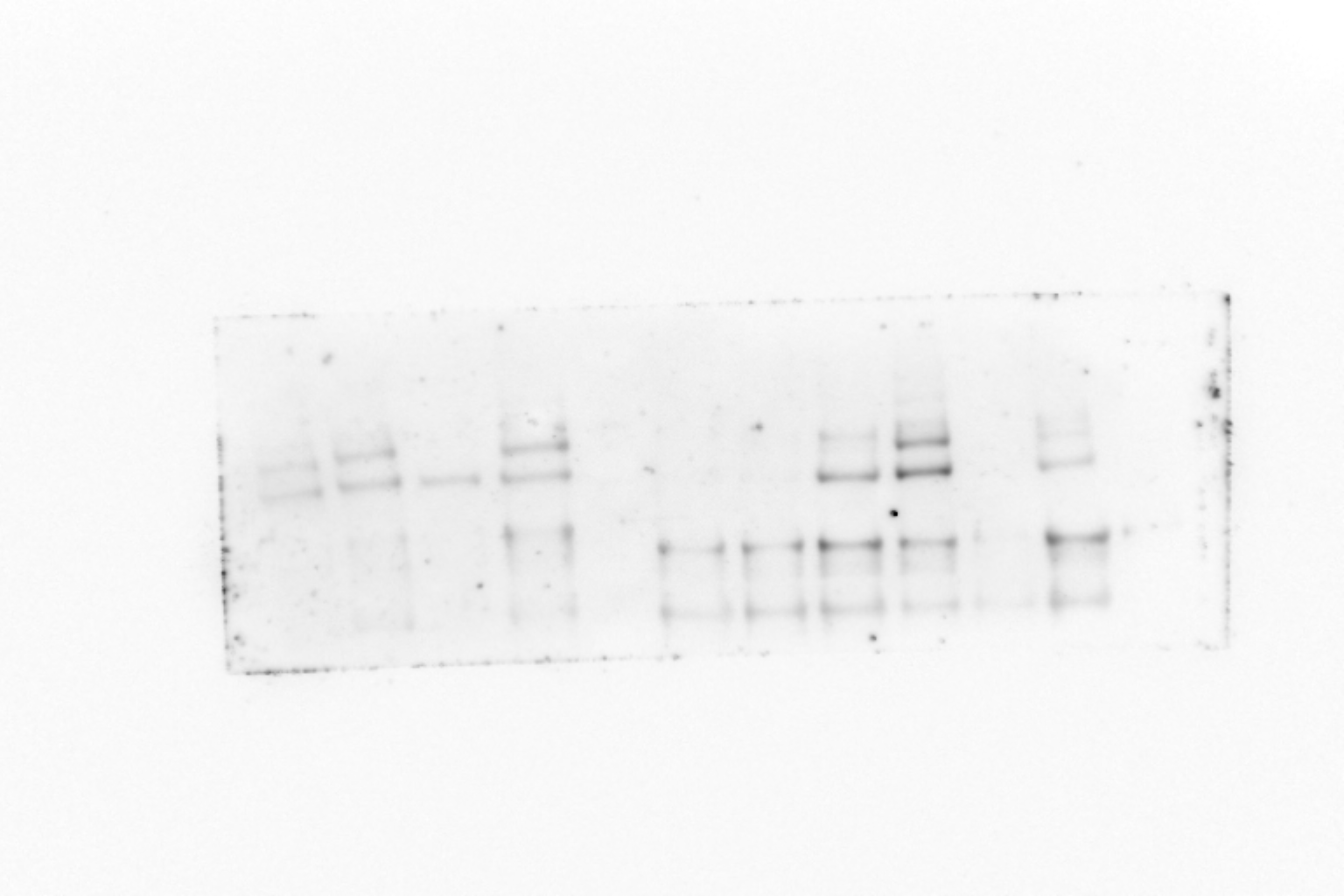

Supplement: Figure 5—source data 5. [file elife-98843-fig5-data5.zip › 5E srs2_Scanner.tif]

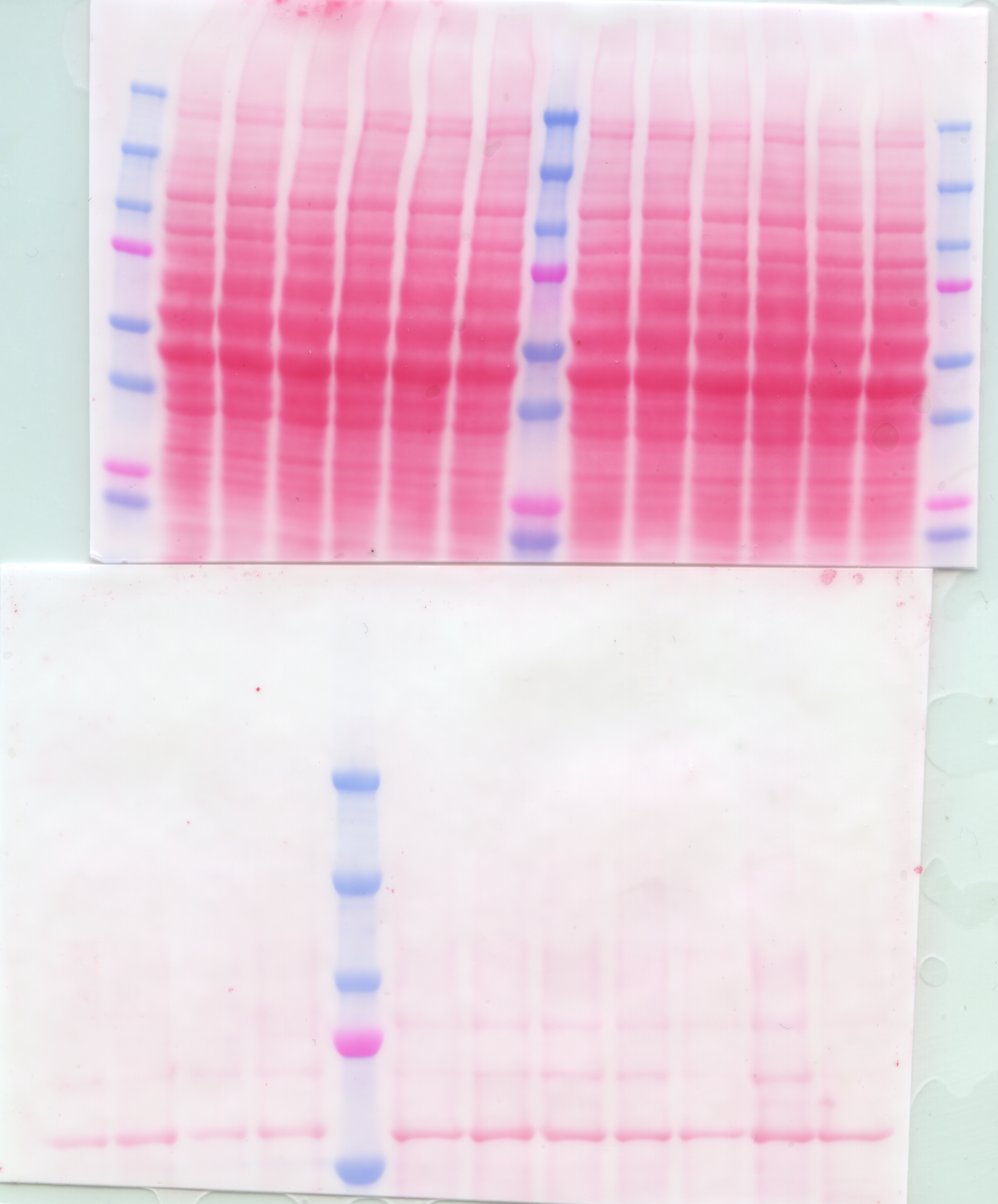

Supplement: Figure 5—source data 5. [file elife-98843-fig5-data5.zip › 5E Ponceau Staining.tif]

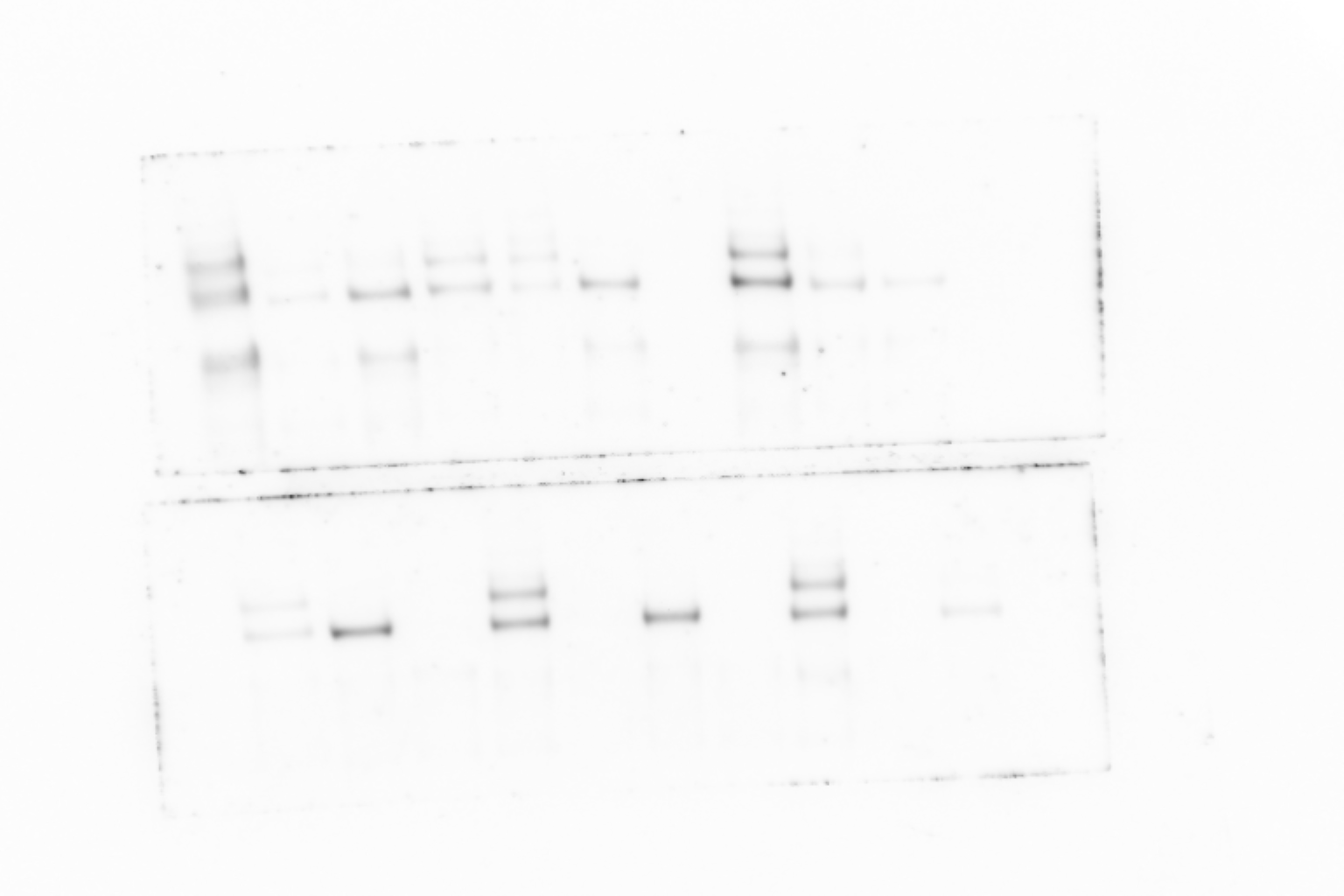

Supplement: Figure 5—figure supplement 1—source data 2. [file elife-98843-fig5-figsupp1-data2.zip › srs2_high_200s.tif]

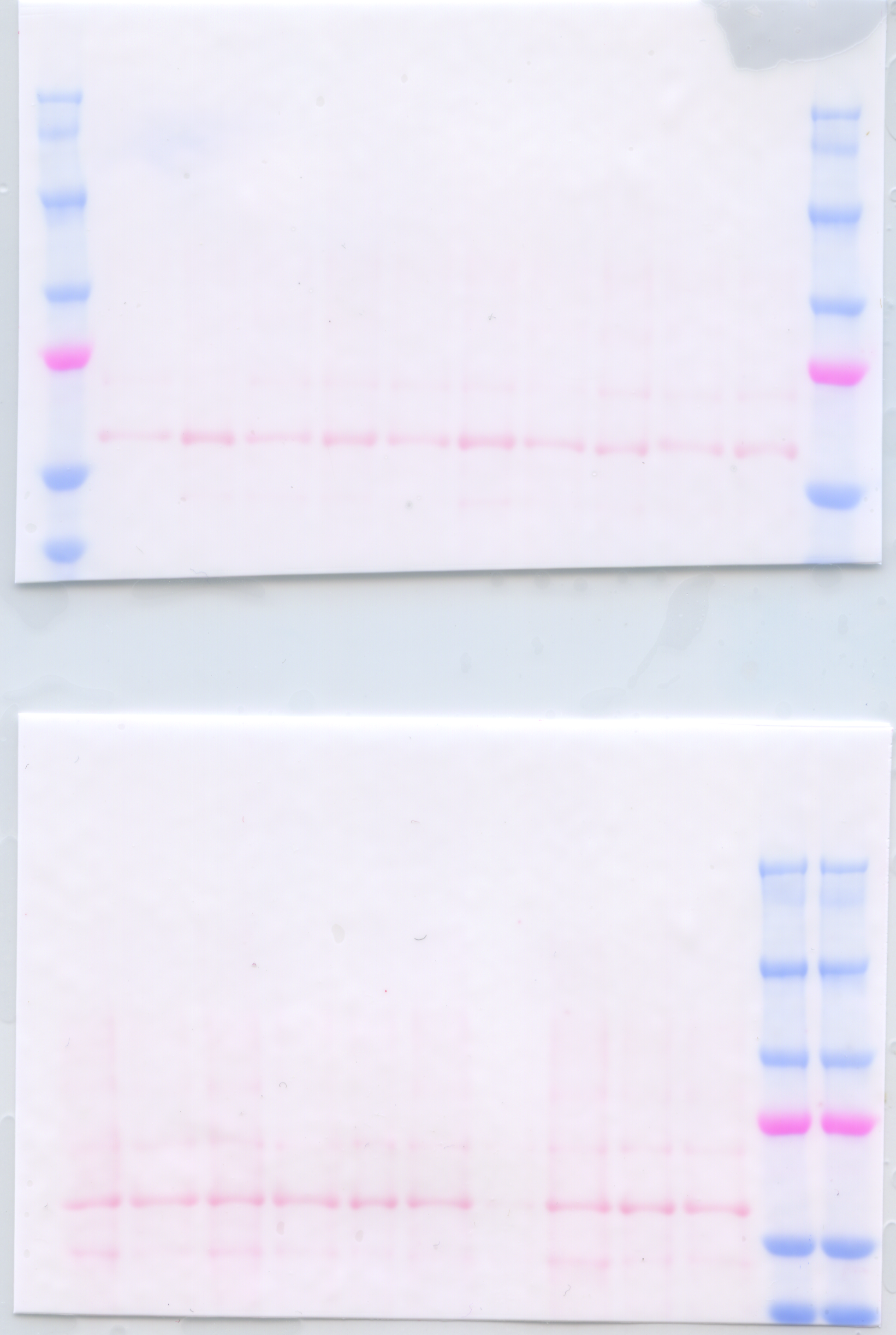

Supplement: Figure 5—figure supplement 1—source data 2. [file elife-98843-fig5-figsupp1-data2.zip › FJY20220602.tif]

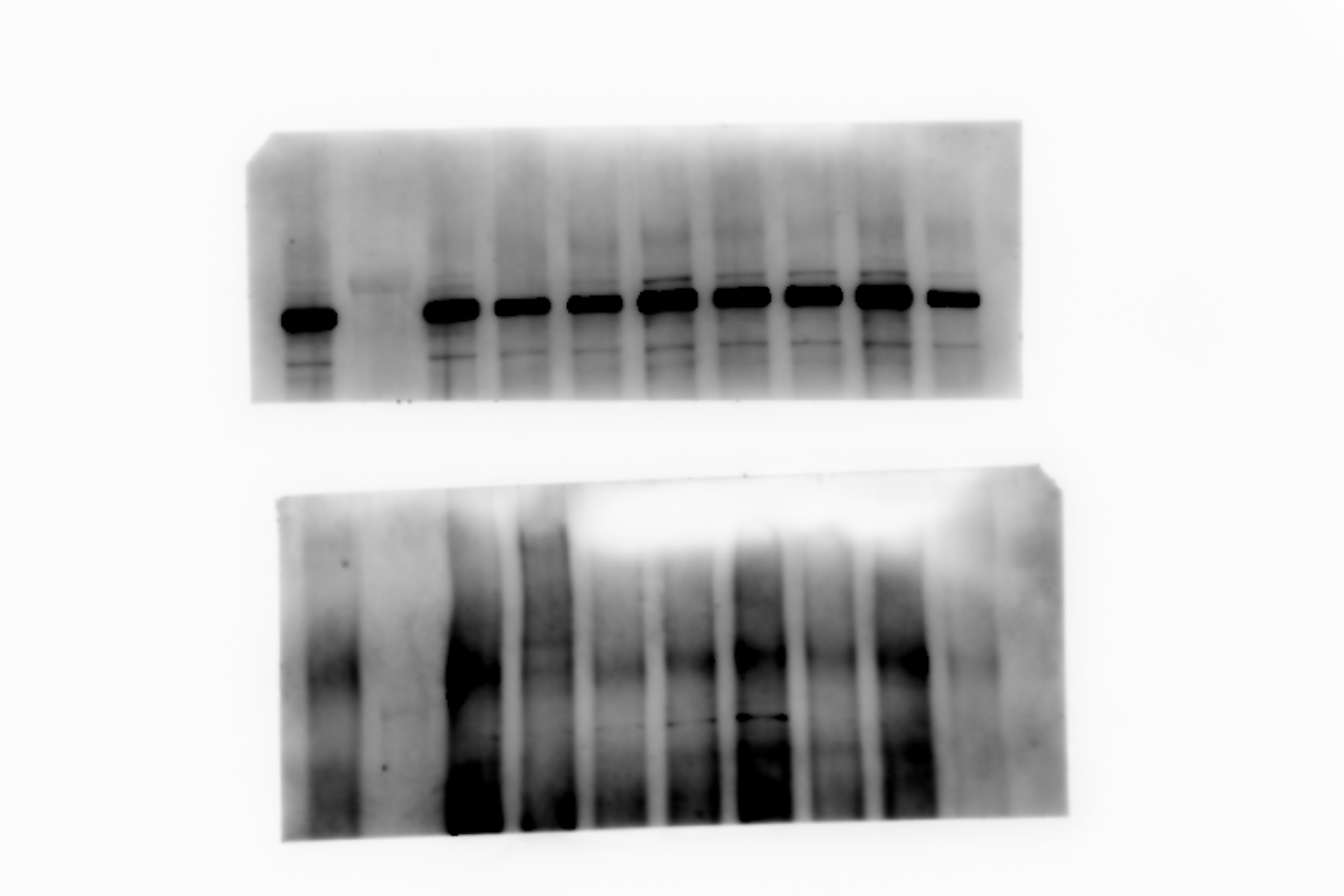

Supplement: Figure 6—source data 2. [file elife-98843-fig6-data2.zip › Mec1-S1964p_Scanner.tif]

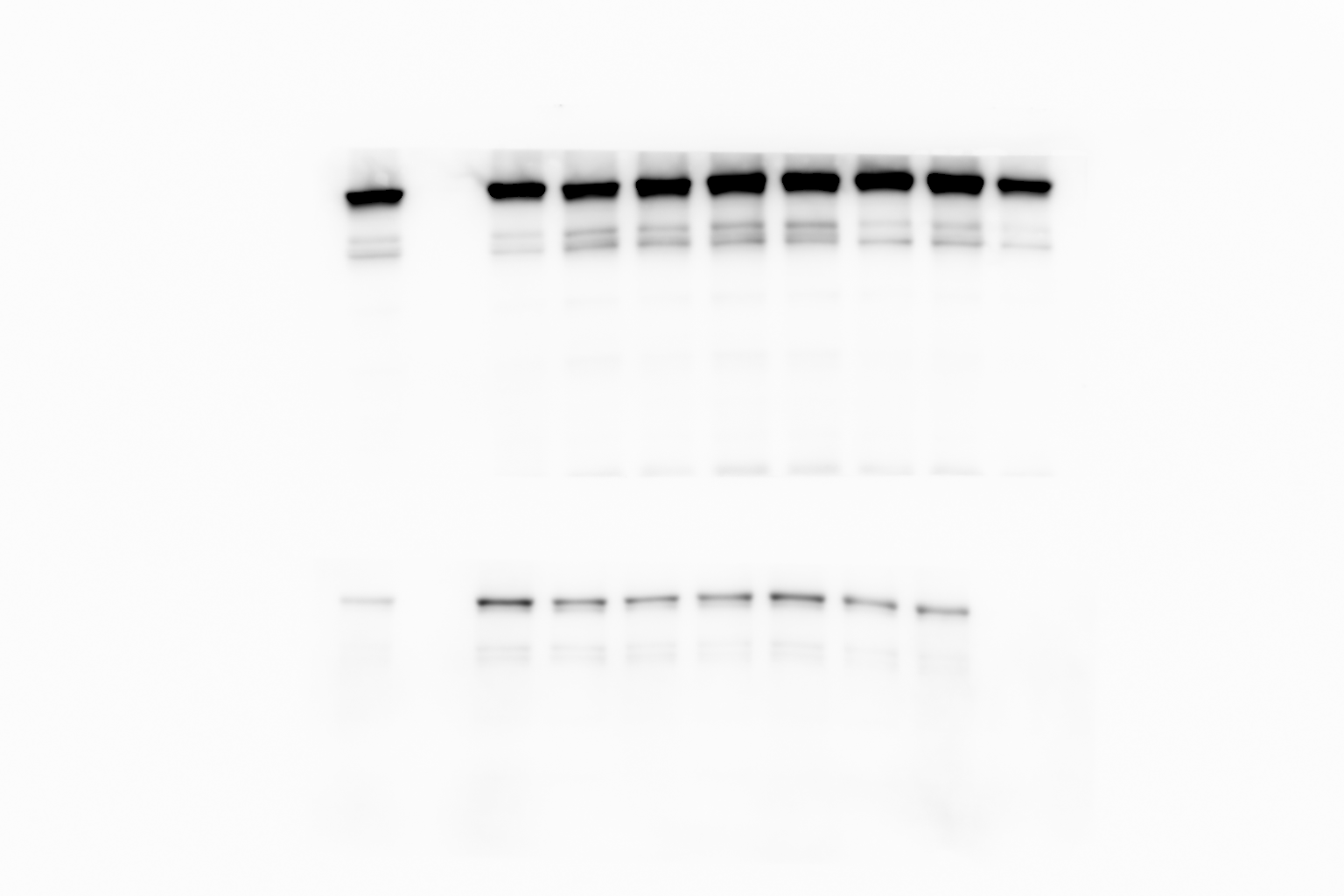

Supplement: Figure 6—source data 2. [file elife-98843-fig6-data2.zip › Ddc2-9myc_Scanner.tif]

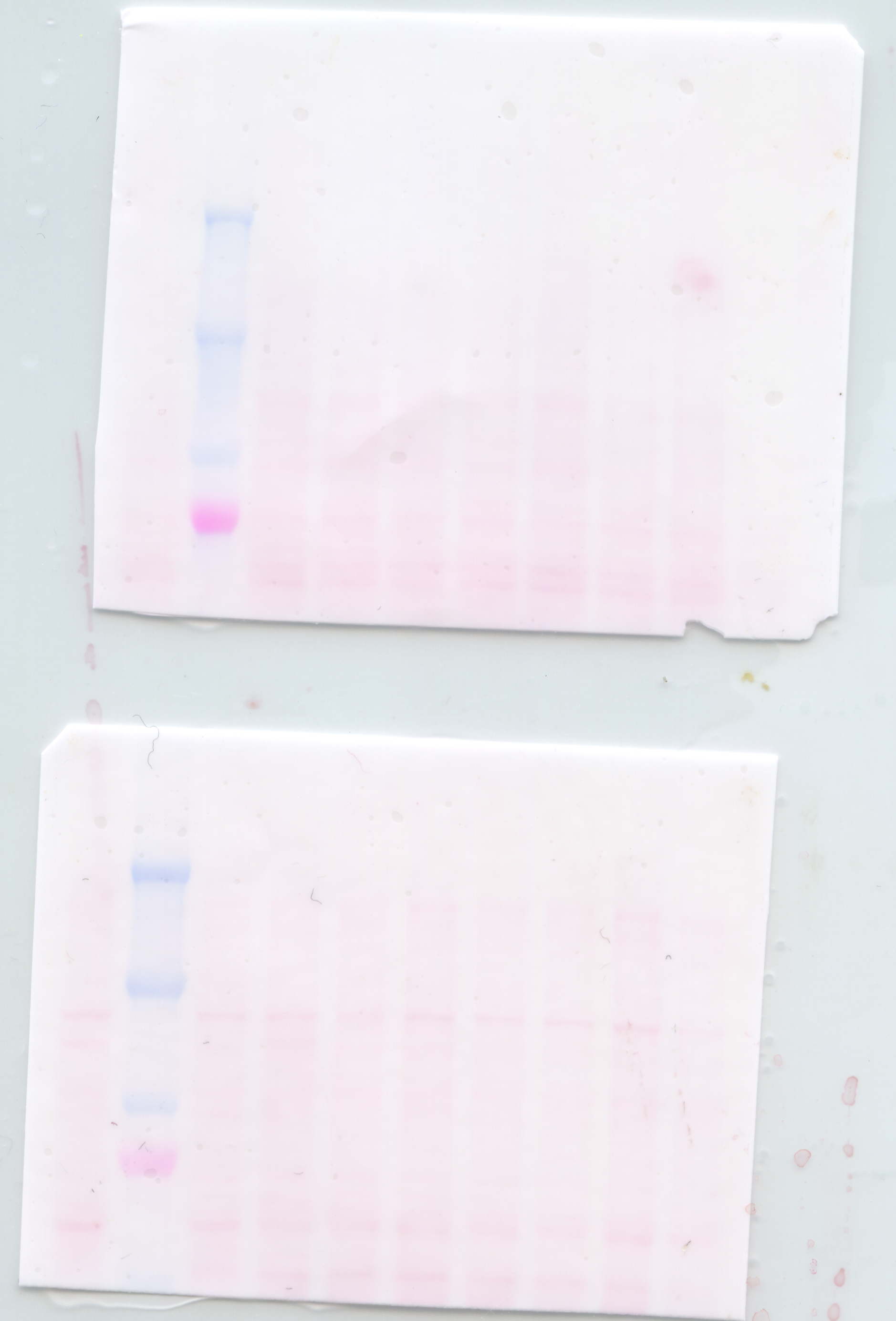

Supplement: Figure 6—source data 2. [file elife-98843-fig6-data2.zip › Ponceau Staining.tif]

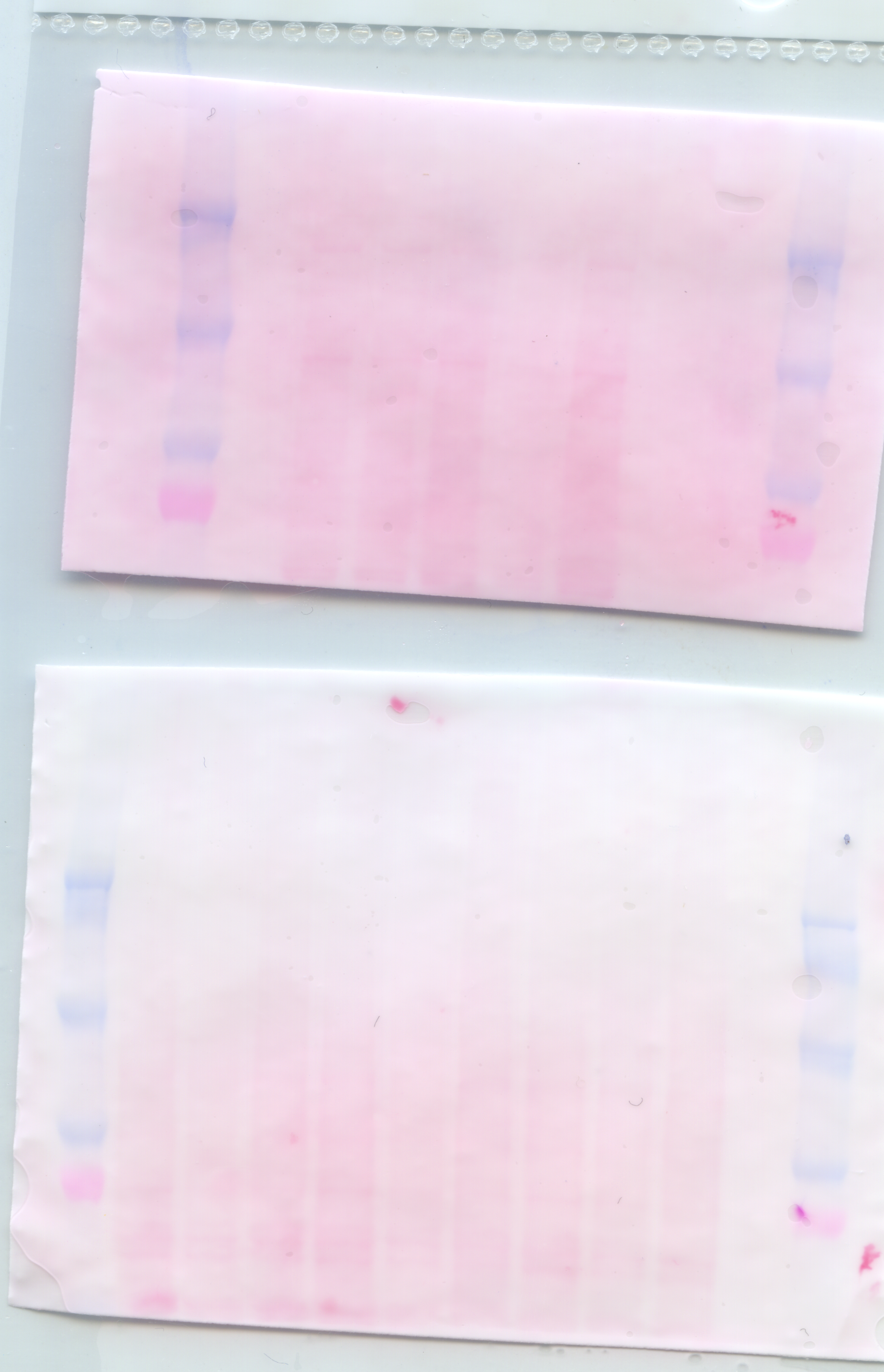

Supplement: Figure 6—figure supplement 1—source data 2. [file elife-98843-fig6-figsupp1-data2.zip › 6Supp A Ponceau Staining.tif]

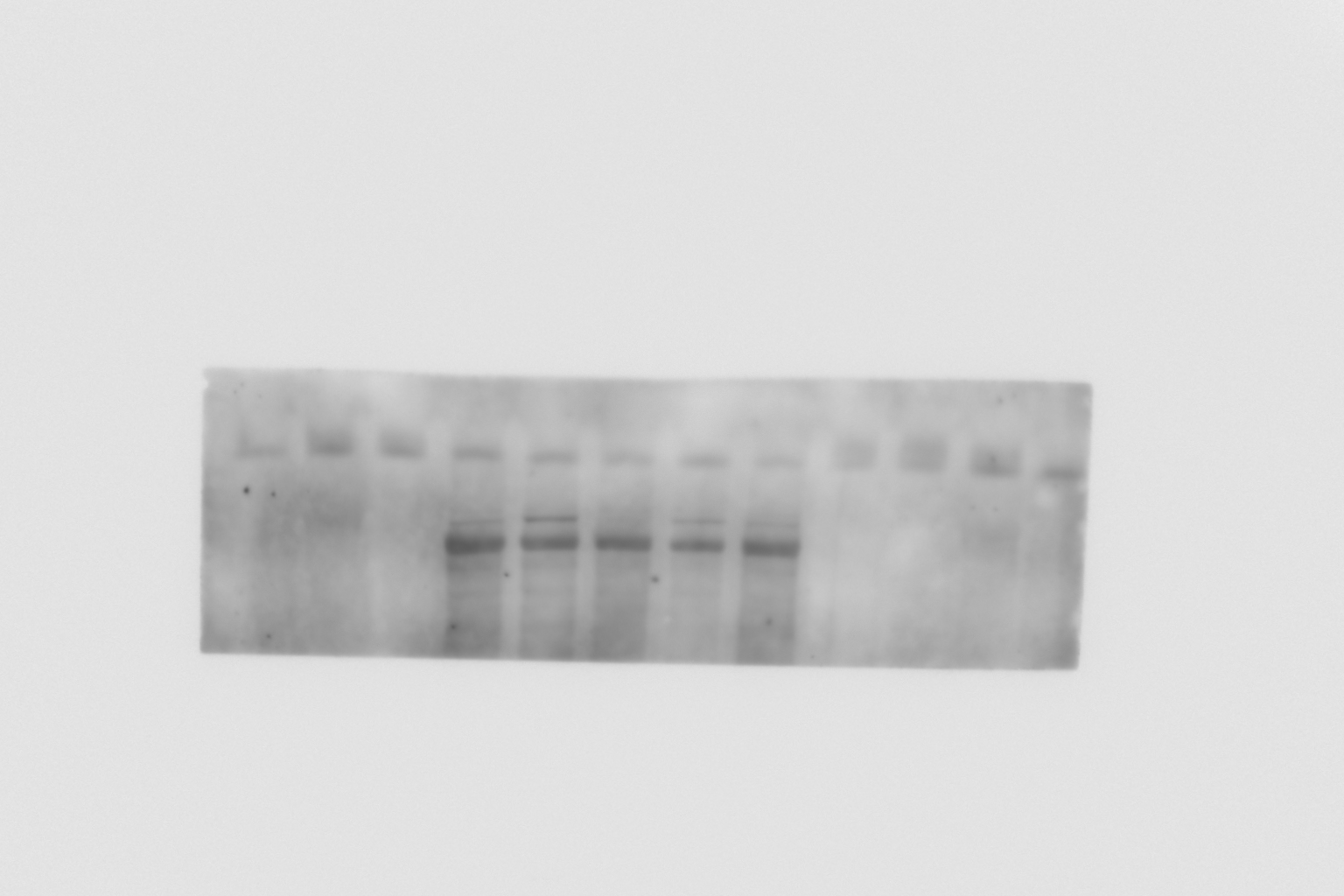

Supplement: Figure 6—figure supplement 1—source data 2. [file elife-98843-fig6-figsupp1-data2.zip › 6Supp A mec1-S1964p_Scanner.tif]

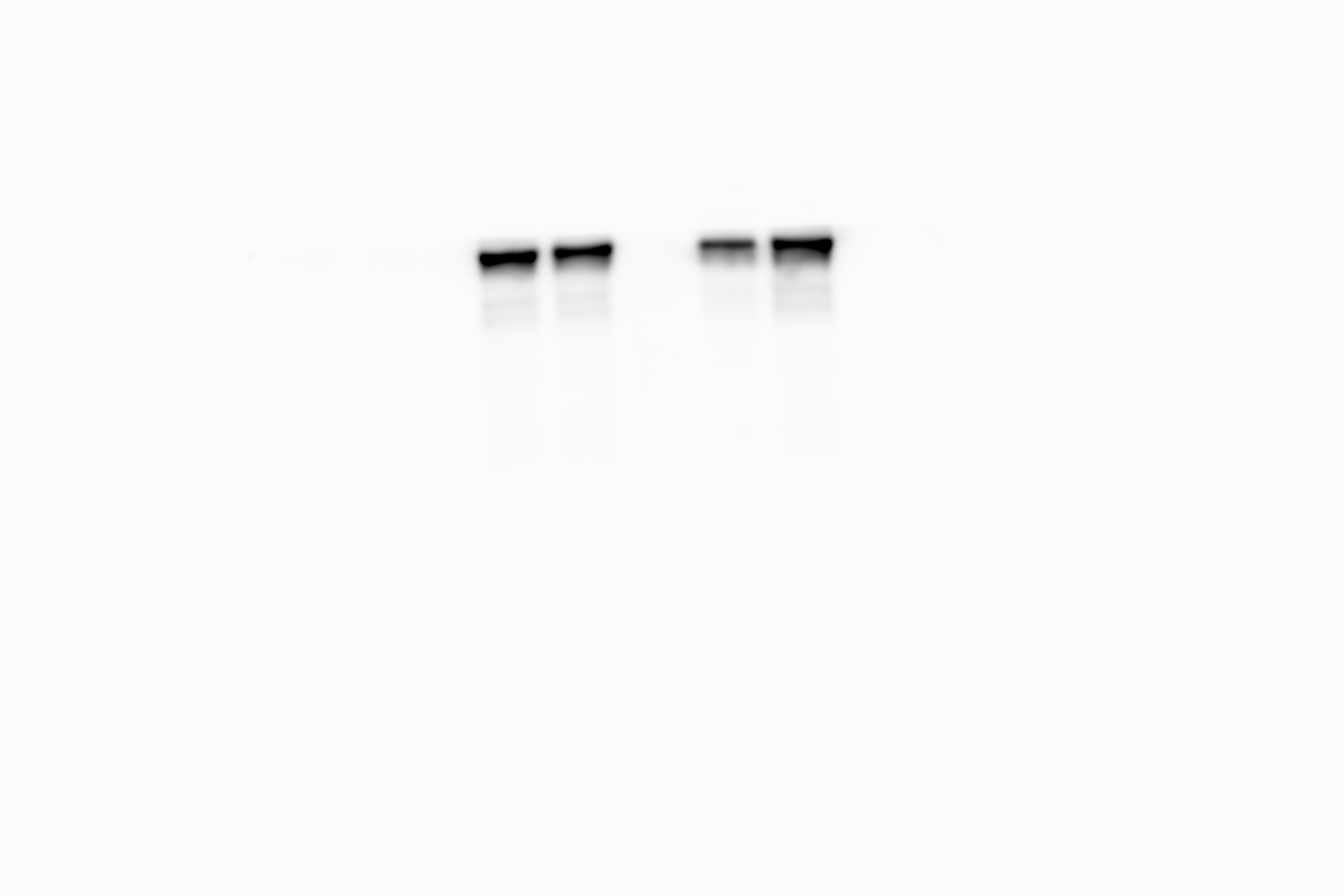

Supplement: Figure 6—figure supplement 1—source data 2. [file elife-98843-fig6-figsupp1-data2.zip › 6Supp A myc_Scanner.tif]

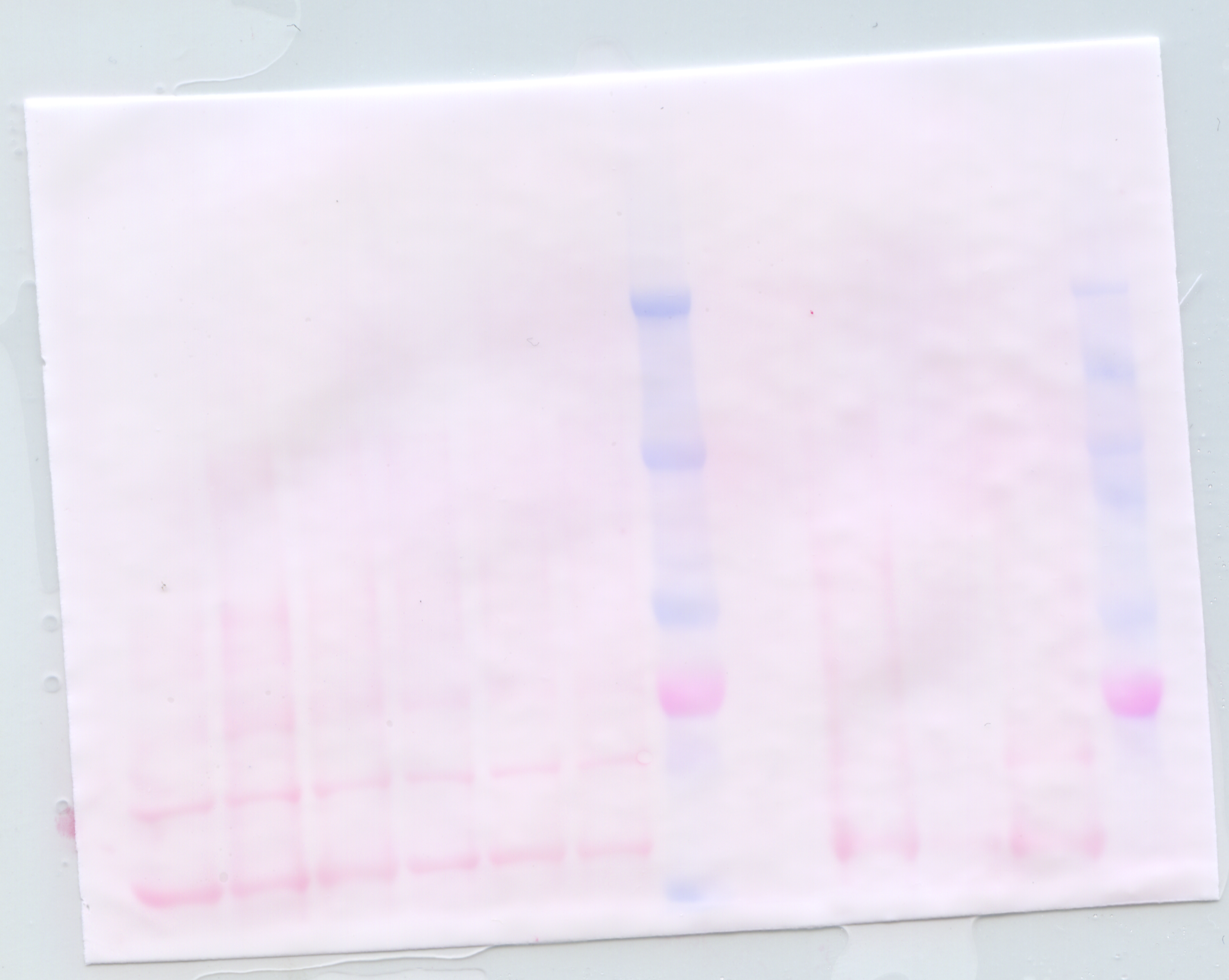

Supplement: Figure 6—figure supplement 1—source data 3. [file elife-98843-fig6-figsupp1-data3.zip › 6Supp D Ponceau Staining.tif]

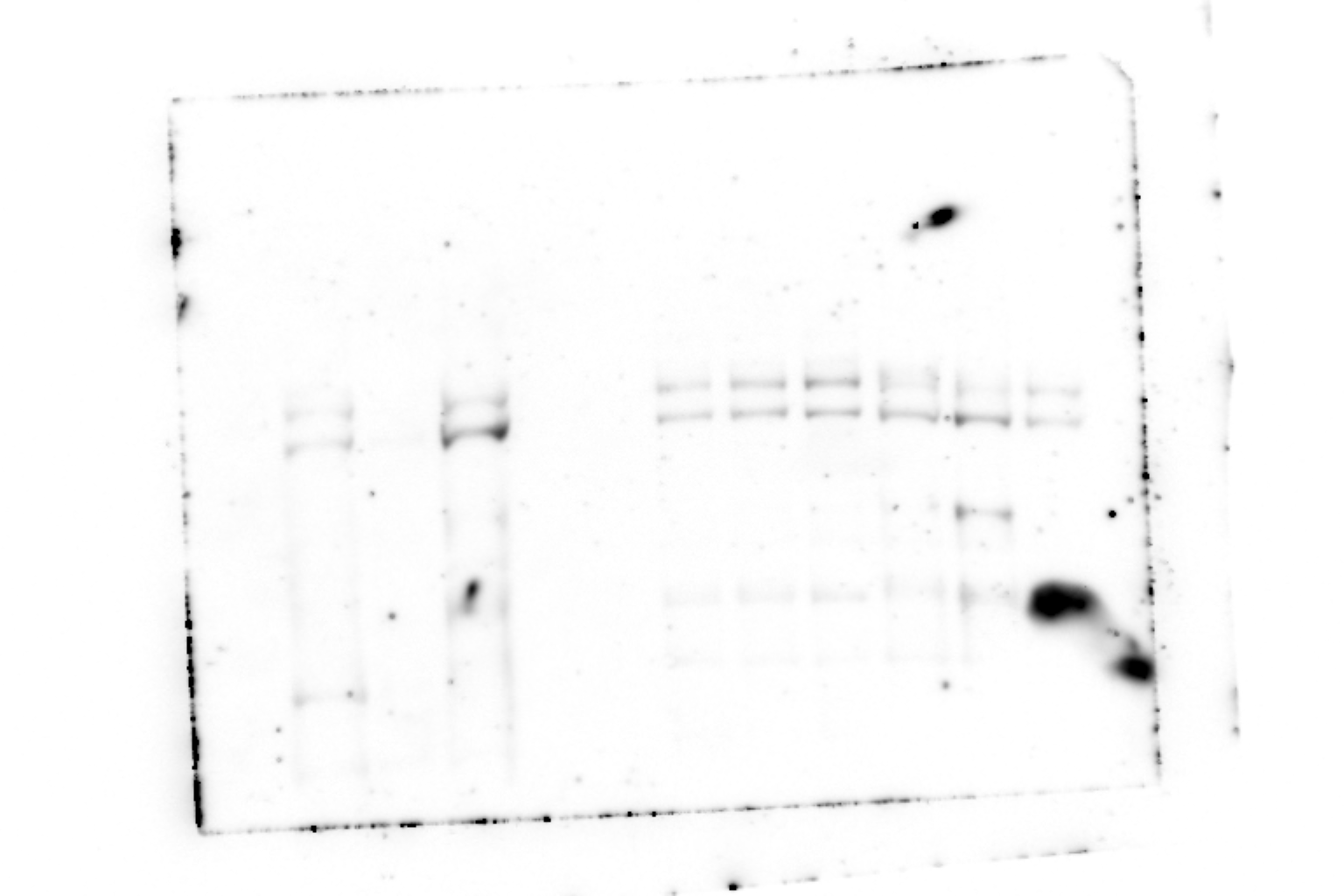

Supplement: Figure 6—figure supplement 1—source data 3. [file elife-98843-fig6-figsupp1-data3.zip › 6Supp D srs2_Scanner.tif]
