# Supplementary material for: Srs2 binding to proliferating cell nuclear antigen (PCNA) and its sumoylation contribute to replication protein A (RPA) antagonism during the DNA damage response
Source: eLife. 2025 Aug 1;13:RP98843. doi: 10.7554/eLife.98843 (PMC12316459; doi:10.7554/eLife.98843)

The blots are overlaid with Ponceau staining to indicate molecular weight. Black squares mark the lanes shown in the figure.

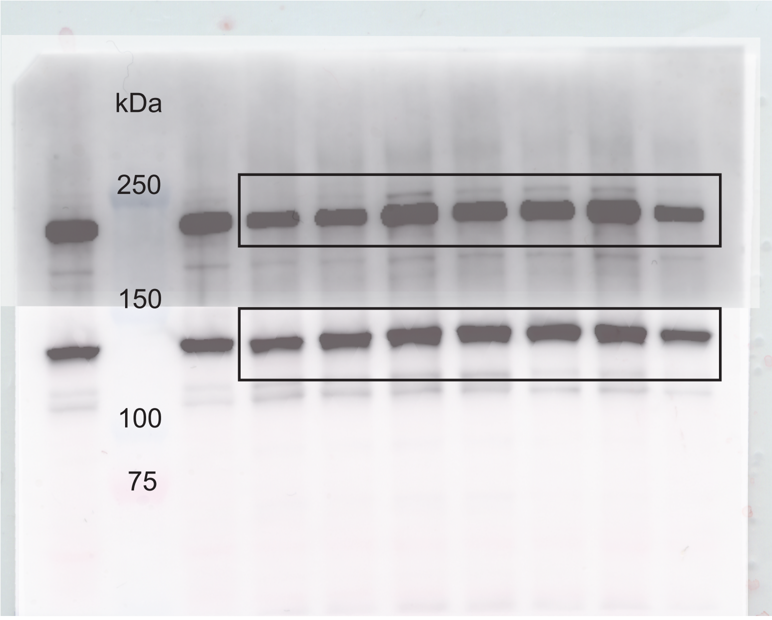

Supplement: Figure 6—source data 1. [file elife-98843-fig6-data1.pdf]
