# Supplementary material for: Srs2 binding to proliferating cell nuclear antigen (PCNA) and its sumoylation contribute to replication protein A (RPA) antagonism during the DNA damage response
Source: eLife. 2025 Aug 1;13:RP98843. doi: 10.7554/eLife.98843 (PMC12316459; doi:10.7554/eLife.98843)
Supplement: Supplementary file 1. [file elife-98843-supp1.docx]

**Supplement Table 1: Strains Sources.**

All strains are derived from W1588-4C, a *RAD5* derivative of W303 (*MATa ade2-1 can1-100 ura3-1 his3-11,15, leu2-3, 112 trp1-1 rad5-535*). Only one strain is listed per each genotype, but at least two independent isolates of each genotype were used in the experiments.

| **Strain** | **Genotype** | **Source** | **Used In Figures** |
| --- | --- | --- | --- |
| G16 | Wild-type W303 *RAD5* strain | Lab collection | 1B, 2C-E, 3B-C, 5C, 6B-D, |
| X8047-14B | *srs2∆* | (Dhingra *et al.*, 2021) | 6C-D |
| X8665-12-22D | *MATa srs2-∆PIM* | This study | 1B, 2C, 3B-C, 6B, |
| X8198-15B | *srs2-SIM^MUT^* | This study | 1B, 2D |
| G1114 | *MATa srs2-7AV::HIS3* | (Saponaro *et al.*, 2010) | 1B, 2D |
| X8650-2-1D | *MATa srs2-3KR::NAT* | This study | 1B, 2C, 3B-C |
| X8659-14A | *MATa srs2-2SA* | This study | 1B, 2D |
| G854 | *MATα srs2-∆Rad51BD(∆(875-902))::HPH* | (Colavito *et al.*, 2009) | 1B, 2D |
| X8047-1B | *MATα rfa1-zm2* | (Dhingra *et al.*, 2021) | 2C, 2C-D, 3B-C, 6B |
| X8650-2C | *MATa srs2-3KR::NAT rfa1-zm2* | This study | 2C, 3B-C |
| X8666-35-6A | *MATa srs2-∆PIM rfa1-zm2* | This study | 2C, 3B-C, 6B |
| X8210-2B | *MATa rfa1-t33* | (Dhingra *et al.*, 2021) | 2E |
| X8745-2A | *MATa srs2-3KR::NAT rfa1-t33* | This study | 2E |
| X8743-13A | *MATα srs2-∆PIM rfa1-t33* | This study | 2E |
| X8198-15A | *srs2-SIM^MUT^ rfa1-zm2* | This study | 2D |
| X8637-6B | *MATa srs2-7AV::HIS3 rfa1-zm2* | This study | 2D |
| X8137-10B | *MATa srs2-∆Rad51BD::HPH rfa1-zm2* | This study | 2D |
| X8660-4B | *MATα srs2-2SA rfa1-zm2* | This study | 2D |
| X9265-4D | *MATa SLX4-TAP::HIS3* | (Wan *et al.*, 2019) | 4A-C |
| X9265-4A | *MATa srs2-∆PIM SLX4-TAP::HIS3* | This study | 4A-C |
| X8956-7C | *MATa slx4^RIM^-TAP::HIS3* | (Wan *et al.*, 2019) | 4A-C |
| X8956-5D | *MATa srs2-∆PIM slx4^RIM^-TAP::HIS3* | This study | 4A-C |
| T1358-2 | *MATa 8His-SMT3::TRP1* | Lab Collection | 5A-B, 5E |
| X8808-2D | *MATa srs2-∆PIM 8His-SMT3::TRP1* | This study | 5A |
| X8795-9D | *MATa srs2-3KR::NAT 8His-SMT3::TRP1* | This study | 5A |
| X8807-6A | *MATa sml1∆::URA3 8His-SMT3::TRP1* | This study | 5E |
| X8746-4D | *MATa sml1∆::URA3 mec1∆::TRP1*  *8His-SMT3::TRP1* | This study | 5E |
| X8807-7A | *MATa sml1∆::URA3 rad53∆::HIS3*  *8His-SMT3::TRP1* | This study | 5E |
| X9055-1D | *MATa DDC2-Myc18::HIS3* | This study | 6A |
| X9225-3A | *MATa mec1-S1964A* | This study | 6B |
| X9225-3C | *MATa mec1-S1964A rfa1-zm2* | This study | 6B |
| X9088-38C | *MATa mec1-S1964A srs2-∆PIM* | This study | 6B |
| X9225-1B | *MATα mec1-S1964A srs2-∆PIM rfa1-zm2* | This study | 6B |
| X8495-5D | *MATa rfa1-S178D* | This study | 6C |
| X8495-2D | *MATa srs2∆::HIS3 rfa1-S178D* | This study | 6C |
| X8494-3B | *MATa rfa1-S178A* | This study | 6C |
| X8494-3A | *MATa srs2∆::HIS3 rfa1-S178A* | This study | 6C |
| X8658-14-22D | *MATa rfa2-3SA* | This study | 6D |
| X8658-14-13C | *MATa srs2∆::HIS3 rfa2-3SA* | This study | 6D |
| X8779-13D | *MATa rfa2-3SD* | This study | 6D |
| X8779-13B | *MATa srs2∆ rfa2-3SD* | This study | 6D |
| X8898-19D | *MATa srs2-∆::HIS3 8His-SMT3::TRP1* | This study | 5-figure supplement 1 |
| X9190-8A | *MATa DDC2-Myc18::HIS3 mec1-S1964A* | This study | 6-figure supplement 1A |
| X9228-2D | *MATa mec1-S1964A srs2-3KR::NAT* | This study | 6-figure supplement 1B |
| X9228-5A | *MATα mec1-S1964A srs2-3KR::NAT rfa1-zm2* | This study |  |
| X9090-9C | *MATa mec1-S1964E* | This study | 6-figure supplement 1C |
| X9091-14A | *MATa mec1-S1964E rfa1-zm2* | This study |  |
| X9090-19A | *MATa mec1-S1964E srs2-∆PIM* | This study |  |
| X9132-15B | *MATa mec1-S1964E srs2-∆PIM rfa1-zm2* | This study |  |
| X9091-13A | *MATa mec1-S1964E srs2-3KR::NAT* | This study |  |
| X9132-10B | *MATα mec1-S1964E srs2-3KR::NAT rfa1-zm2* | This study |  |
| X9137-3B | *MATa mec1-S1964A 8His-SMT3::TRP1* | This study | 6-figure supplement 1D |
| X9136-2B | *MATa mec1-S1964E 8His-SMT3::TRP1* | This study |  |

**Supplement Table 2: CRISPR-Cas9 editing details for yeast mutants.**

| **Mutated gene** | **Cas9 targeting sequence (PAM)** |
| --- | --- |
| Srs2 | AAAGTCTAAGAGAGGTGACA(AGG) |
| Rfa1 | AGTGCGACACCAATAATGCA(AGG) |
| Rfa2 | TGAAGTGTTGACGCATCACT(TGG) |
| Mec1 | GCACTAGAAACTAGATCATG(AGG) (Memisoglu et al., 2019) |
